# Insights on TAM Formation from a Boolean Model of Macrophage Polarization Based on In Vitro Studies

**DOI:** 10.3390/cancers12123664

**Published:** 2020-12-07

**Authors:** Malvina Marku, Nina Verstraete, Flavien Raynal, Miguel Madrid-Mencía, Marcin Domagala, Jean-Jacques Fournié, Loïc Ysebaert, Mary Poupot, Vera Pancaldi

**Affiliations:** 1 INSERM, Centre de Recherches en Cancérologie de Toulouse, 2 Avenue Hubert Curien, 31037 Toulouse, France; nina.verstraete@inserm.fr (N.V.); flavien.raynal@inserm.fr (F.R.); miguel.madrid-mencia@inserm.fr (M.M.-M.); marcin.domagala@inserm.fr (M.D.); jean-jacques.fournie@inserm.fr (J.-J.F.); ysebaert.loic@iuct-oncopole.fr (L.Y.); mary.poupot@inserm.fr (M.P.); 2 Université III Toulouse Paul Sabatier, Route de Narbonne, 31330 Toulouse, France; 3 Service d’Hématologie, Institut Universitaire du Cancer de Toulouse-Oncopole, 31330 Toulouse, France; 4 Barcelona Supercomputing Center, Carrer de Jordi Girona, 29, 31, 08034 Barcelona, Spain

**Keywords:** Boolean model, tumour associated macrophage, macrophage polarization, nurse-like cells, chronic lymphocytic leukaemia

## Abstract

**Simple Summary:**

The recent success of immunotherapy treatments against cancer relies on helping our own body’s defenses in the fight against tumours, namely reinvigorating the cancer killing action of T cells. Unfortunately, in a large proportion of patients these therapies are ineffective, in part due to the presence of other immune cells, macrophages, which are mis-educated by the cancer cells into promoting tumour growth. Here we start from an existing model of macrophage polarization and extend it to the specific conditions encountered inside a tumour by adding signals, receptors, transcription factors and cytokines that are known to be the key components in establishing the cancer cell-macrophage interaction. Then we use a mathematical Boolean model applied to a gene regulatory network of this biological process to simulate its temporal behaviour and explore scenarios that have not been experimentally tested so far. Additionally, the KO and overexpression simulations successfully reproduce the known experimental results while predicting the potential role of regulators (such as STAT1 and EGF) in preventing the formation of pro-tumoural macrophages, which can be tested experimentally.

**Abstract:**

The tumour microenvironment is the surrounding of a tumour, including blood vessels, fibroblasts, signaling molecules, the extracellular matrix and immune cells, especially neutrophils and monocyte-derived macrophages. In a tumour setting, macrophages encompass a spectrum between a tumour-suppressive (M1) or tumour-promoting (M2) state. The biology of macrophages found in tumours (Tumour Associated Macrophages) remains unclear, but understanding their impact on tumour progression is highly important. In this paper, we perform a comprehensive analysis of a macrophage polarization network, following two lines of enquiry: (i) we reconstruct the macrophage polarization network based on literature, extending it to include important stimuli in a tumour setting, and (ii) we build a dynamical model able to reproduce macrophage polarization in the presence of different stimuli, including the contact with cancer cells. Our simulations recapitulate the documented macrophage phenotypes and their dependencies on specific receptors and transcription factors, while also unravelling the formation of a special type of tumour associated macrophages in an in vitro model of chronic lymphocytic leukaemia. This model constitutes the first step towards elucidating the cross-talk between immune and cancer cells inside tumours, with the ultimate goal of identifying new therapeutic targets that could control the formation of tumour associated macrophages in patients.

## 1. Introduction

As all living cells, macrophages perceive and respond to intra- and extracellular signals in order to maintain their functions (endocytic, phagocytic and secretory, for example) by displaying a wide spectrum of specific phenotypes (polarizations) in different inducer environments. Based on their activity and the expression of specific proteins, markers and chemokines, two major subsets of macrophages have been identified, namely classically activated macrophages (M1) exhibiting a pro-inflammatory response, and alternatively activated macrophages (M2, themselves subdivided into 4 subclasses: M2a, M2b, M2c, M2d [1,2,3]) exhibiting an anti-inflammatory response. Additionally, multiple studies support the idea that M1 and M2 macrophages represent, in fact, the extremes of a continuous polarization spectrum of cells deriving from the differentiation of monocytes [4]. Their plastic gene expression profile is determined by the type, concentration and duration of exposure to the polarization stimuli in an inflammatory environment [3,5,6,7,8].

Macrophages are also found inside tumors, as part of the tumor micro-environment (TME), a complex collection of cells that are found surrounding cancer cells including also other immune cells, such as lymphocytes and neutrophils, as well as other normal cells. In many tumors, infiltrated macrophages display mostly an M2-like phenotype, which provides an immunosuppressive microenvironment. In cancer, these *tumor associated macrophages (TAMs)* secrete several cytokines, chemokines and proteins which promote tumor angiogenesis, growth and metastasis [9,10,11,12]. Interestingly, it was observed that in established tumors, signals originating from cancer cells can cause phenotypic shifts in macrophages, leading to alternative functions that do not correspond to either M1 or M2 phenotypes [13]. Several studies have demonstrated that TAMs directly suppress CD8${}^{+}$ T cell activation in vitro [14,15,16,17]. Mechanisms that orchestrate this process, either directly or indirectly, remain unclear [18] and warrant further exploration due to macrophages’ important impact on tumor progression.

The TME can be defined as an ecological system given the presence of diverse types of cells that interact in specific ways with each other. To name a few, cytotoxic T lymphocytes can attack cancer cells and kill them, while macrophages can phagocyte apoptotic or dead cells. However, complex feedback relations exist between the signals produced by some cell types and the phenotypic transitions that are induced by these signals in other cells. For example, due to the high density of leukemic B cells, monocytes residing in lymph nodes from CLL patients tend to differentiate into a pro-tumoral state, which is able in turn to promote survival of cancer cells through the secretion of anti-apoptotic signals, rather than eliminating them. These complex interrelations between cells are typical of predator-prey systems in ecology and can be modelled using similar approaches in which the possible final states (attractors) of the system define which populations will dominate [19].

In any given environment, the cellular processes that determine a cell’s phenotype consist in a cascade of interactions, which can be represented as a regulatory network, in which nodes represent proteins, enzymes, chemokines, etc., while the connections represent the type (activation or inhibition) and direction of interactions of different types (transcriptional and post-translational activations). Network modelling has found numerous applications in studying the structure and dynamic behaviour of different biological systems in response to environmental stimuli and internal perturbations [20,21,22,23]. Several computational models of different pathways involved in the inflammatory immune response have been previously published, such as: continuous, logical and multi-scale models of T cell differentiation [24,25,26], logical models of macrophage differentiation in pro- and anti-inflammatory conditions [27], multi-scale models of innate immune response in tumoral conditions [28], etc.

Macrophages are extremely plastic cell types, whose phenotypes can easily switch depending on conditions. Since the specific macrophage state that protects cancer cells from undergoing apoptosis is fundamental in the development of resistance to treatments in CLL and solid tumors, we turned to study it as a polarization state. An important computational model of macrophage polarization was able to detect 3 different M2 subgroups of macrophages, as a result of various combinations of pro- and anti-inflammatory extra-cellular signals [27], using exclusively literature-based knowledge of the intra-cellular regulatory interactions and pathways involved in the polarization process. In a more recent work, Ramirez et al. [29] used temporal expression profiles of in vitro macrophage cytokines to infer logical models of macrophage polarization (M1 and 3 subcategories of M2: M2a, M2b and M2c) in the presence of different stimuli. Nevertheless, many important questions remain to be explored regarding the polarization states, especially in a tumor setting. More specifically, it is important to identify the pathways involved in TAM formation and to understand to what extent the macrophage plasticity facilitates this process inside a tumor. On the other hand, despite the wealth of quantitative information from bulk and single-cell sequencing datasets, the inference of regulatory networks based on experimental data remains a difficult challenge, with most approaches proposing a combination of both literature- and data-driven methods [29,30,31,32].

In Chronic Lymphocytic Leukemia (CLL), a B-cell malignancy in which patients accumulate large quantities of malignant CLL cells in their lymph nodes, an interesting ecology of cancer cells and immune cells is established. CLL cells are able to educate surrounding monocytes, through direct contact and cytokine signals, turning them into TAMs, which in this disease are referred to as Nurse Like Cells (NLCs) [33]. NLCs are derived from CD14${}^{+}$ monocytes and are characterised by a distinct set of antigens (CD14lo, CD68hi, CD11b, CD163hi) [34,35]. Moreover, NLCs express stromal-derived-factor-1alpha, a chemokine which promotes chemotaxis and activates mitogen activated protein kinases, ultimately leading to more aggressive cancers and better survival of these cells in vitro. Through direct contact, the NLCs are able to protect the cancer CLL cells from apoptotic signals, and stimulate environment mediated drug resistance. Interactions between NLCs and CLL cells appear to be mediated by the B cell receptor, which, when stimulated, activates production of CCL3/4, initiating the recruitment of other cells, including CD4${}^{+}$ T cells and more NLCs. Another pathway that has been associated with NLCs and TAMs more in general is that of CSF-1 (MCSF). Patients with high expression of this factor usually show faster CLL progression and this gene was implicated in the production of NLCs. Also the more M1- or M2-like profile of NLCs in specific patients correlates with active and controlled disease, respectively. Analyses of the transcriptomic profile of NLCs suggest their high similarity to the macrophage M2 profile described in solid tumors, which makes studying the formation of NLCs all the more relevant in the quest of controlling TAMs in other malignancies.

The main characteristics of these 3 types of macrophages are given in Table 1. Considering the close phenotypic similarity between M2 and NLC/TAM macrophages, we consider that the NLC/TAM components should include the M2 ones. Here we indicate in blue the components that have been used in our model and in bold the ones that were taken as signature components for each phenotype. A schematic diagram of M1 and M2 (with 4 subcategories) macrophages can be found in [2], whereas a short description of the profiles for the main macrophage phenotypes is given in Appendix A. More detailed explanations of the mechanisms, pathways and components involved in the polarization process can be found in the cited papers and the references therein.

NLC formation can be studied through an in vitro system in which co-cultures of monocytes and patient-derived CLL cells can be established to produce NLCs in absence of any other cell type. This system is particularly suited to mathematical modelling, as experimental conditions are well controlled and the cell types present are limited to monocytes/macrophages and cancer cells, without the confounding effects of other immune or healthy cells.

Boolean models are discrete dynamical models, in which each component (gene, transcription factor, chemokine, cytokine, receptor, etc.) is associated with a discrete (binary) variable, representing its concentration, activity or expression. Despite the complex processes relating the transcription of a gene into an mRNA and its subsequent translation into a protein with possibly post-translational modifications, in this paper we consider a single node for gene, mRNA and protein, such that a link between two transcription factors signifies that one of them affects transcription of the gene coding for the other.

The future states of each component are determined by the current states of its regulators, as given by a Boolean function that represents the regulatory relationships between the components according to the logic operators AND, OR and NOT. The state of the system at each time point is given by a binary vector, in which each element represents the state of the corresponding component (ON/OFF) [24,26]. Starting from an initial state, as time passes the system will follow a trajectory of states reaching one of many attractors that can be a single stable state (fixed point) or a set of recurrent states (limit cycle). Attractors usually represent specific phenotypes, such as cellular differentiated states, cell cycle states, etc. Despite their coarse-grained description, Boolean models have been successfully used to capture real-world biological features such as, for example, the mechanisms of cell fate decision [45], hierarchical differentiation of myeloid progenitors [46], dynamical modelling of oncogenic signalling [47], among many other applications [48,49,50]. One of their main advantages is the simplicity of performing in-silico experiments simulating a variety of mutant and knockout conditions, and the possibility of obtaining qualitative or semi-quantitative results without requiring experimentally derived parameter values, as needed by differential equations. Starting from a pathway diagram describing a biological process, and adding logic rules, Boolean models allow us to model the process, uncover the main regulators, and run simulations.

Understanding the mechanisms of TAM formation is of particular interest because of their pro-tumoral activity which hampers T cell cytotoxic activity. In this study, we therefore follow two lines of enquiry: (i) we use a literature and data-driven approach based on an in vitro model of NLC formation to reconstruct a macrophage polarization regulatory network, (ii) we implement a Boolean model of monocyte differentiation into NLC simulating these in vitro cultures.

## 2. Results

In this section we describe a model of differentiation of monocytes into macrophages of different polarization states, including the Nurse Like Cell state which is produced in in vitro experiments of monocyte co-culture with cancer cells. We describe how previous models of macrophage polarization were extended to account for this new cell type and compare results of our simulations with existing experimental evidence of the effect of treatments and mutants on the polarization spectrum. Section 2.1 will describe the reconstruction of the regulatory network underlying macrophage polarization while Section 2.2 will explain how Boolean rules were applied to this regulatory network and the dynamics of the resulting model.

### 2.1. Reconstruction of the Regulatory Network Leading to NLC Formation

To reconstruct the gene regulatory network (GRN) governing the formation of NLCs, we started from a previous macrophage polarization GRN [27] and extended it in order to include specific extracellular signals found in the Chronic Lymphocytic Leukaemia (CLL) context and other intra-cellular components involved in NLC formation. The network extension was based on extensive literature review and transcription factor (TF) activities estimation for each phenotype.

Starting from the model in [27], we further added some nodes based on literature (signals: M-CSF, HMGB1; receptors: M-CSFR, RAGE; intracellular components: IRF5, EGF, TNFα, TGFβ, HIF1α, Table S1a) which describe the mechanisms involved in the establishment of the interaction between NLC and CLL, such as receptor–ligand interaction (M-CSF - M-CSFR, HMGB1 - RAGE [37]) that are frequently present in tumoral environments. We then used public transcriptomics data for monocytes and M1, M2 and NLCs to calculate the TF activities in each phenotype. In these experiments, monocytes were either not induced, induced to mature to macrophages with M-CSF and further activated with Interferon gamma and LPS (M1) or IL-4 (M2) [51,52]. The complete analysis of TF estimation on microarray gene expression datasets was performed using the Dorothea R package and Viper [53] and ISMARA [54] (see Methods, Section 5.3). The list of most active TFs for each phenotype (M1, M2 and NLC) from both methods can be accessed in Tables S2a–d and S3a–d.

For NLCs, we identified specific TFs using a set of 19 microarray expression profiles we have generated using an in vitro co-culture experiment in which NLCs are differentiated from monocytes upon contact with B cells from a CLL patient [55]. Interestingly, we could find a few TFs with higher activities in NLC than in M1 and M2 (Tables S2a, S2d, S3a and S3d). Of this set, we were particularly interested in HIF1α and IRF5, which we added to our model (Table S1a). HIF1α is known to be linked to the pro-tumoral activity of NLC through the HIF1α-dependent activation of CXCL12, an important cytokine secreted by NLCs [52,56]. We thus considered this TF among the key regulators for the NLC phenotype. On the other hand, although showing a higher estimated activity in NLC compared to M1 and M2, IRF5 is known to lead to M1-polarization by IL10 repression and TNFα up-regulation [2,57], which is why it was included as a key component in the model but not in any signature (see Section 4 section for further details).

**Figure 1:**
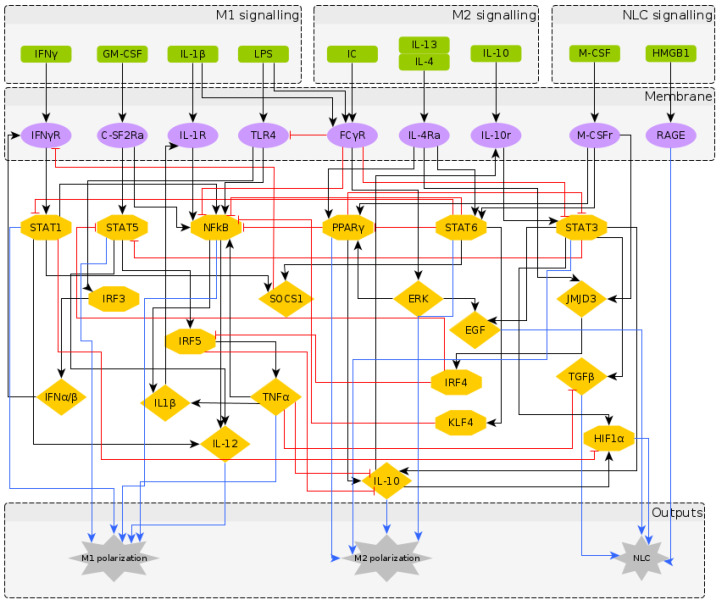
The regulatory network of macrophage polarization: Nodes in green represent the extra-cellular signals, classified as M1, M2 and NLC inducers; nodes in purple represent receptors in the macrophage membrane, usually activated upon contact with cells in the outer environment; nodes in yellow represent the transcription factors and chemokines involved in the polarization process, as an intermediate step or as an output. The diamond nodes represent cytokines whereas the hexagonal nodes represent transcription factors. The interactions between components can be either activation (black) or inhibition (red), while the blue arrows represent the markers for each polarization state.

The reconstructed regulatory network of macrophage polarization is given in Figure 1. It contains 10 extracellular signals, 30 intra-cellular components, most of them being TFs and interleukins, and 3 outputs, which are used as readouts, namely M1 polarization, M2 polarization and NLC. Pathway enrichment analysis [58] showed that most of the components are involved in the JAK-STAT signalling pathway, pathways related to cancer, Th17 cell differentiation, cytokine receptor interaction and other inflammatory conditions. Having obtained a regulatory network that has the potential to describe the formation of NLCs, we turned to literature to assign to each network node a Boolean rule for its activation.

### 2.2. A Boolean Model of Macrophage Polarization

To understand the temporal behaviour of the regulatory system in Figure 1, we model its dynamics by a system of Boolean functions, each of which describes the regulation of the expression of each component. Starting from the Boolean model in [27], we wrote the Boolean functions for the additional nodes based on a supervised literature-based method, using the expression profiles of the macrophage phenotypes taken from the literature (in which most markers of the different macrophage types are protein surface markers). It is important to note that while the NOT operator can be inferred from the regulatory interactions, the AND and OR operators require deeper knowledge of dependent or independent action of regulators on a specific target. We performed a significant number of simulations to explore a wide range of combinations of AND and OR operators and to select the operators’ combination that would identify distinct macrophage phenotypes in the attractor space. The Boolean functions for each intracellular component are given in Table 2. The numerical simulations were performed considering all the possible initial intracellular conditions and combinations of stimuli, while applying the synchronous updating method to calculate the system’s attractors (Section 5.1).

The simulation results show that the system reaches 1384 fixed point attractors, while other cyclic attractors of length 2 and 3 were also present. For our scope, in the following paragraphs we focus only on the fixed point attractors. It is important to note that fixed point attractors are time invariant, i.e., the number of fixed points is not affected from the updating method chosen, while the number of cyclic attractors and their characterisitcs (period, basin of attraction) depend on the updating method (Section 5.1). Here and throughout the paper, we will refer to an attractor as the binarized expression profile which we assign to a polarization state (or a phenotype). To attribute the attractors to certain phenotype categories, we removed all the input nodes (extracellular signals) from the attractors, thus reducing the attractors’ space to 214 fixed points.

### 2.3. Phenotype Identification through Interpretation of the Attractors

The large attractors’ space raises the challenge of interpreting its biological meaning. To categorize the attractors in specific polarization states, two different methods were used: (1) a supervised literature-based method using the expression profiles of the macrophage phenotypes taken from the literature, and (2) an unsupervised method grouping attractors based on their similarity and then applying clustering algorithms to assign them to specific phenotypes.

#### 2.3.1. Interpreting Attractors based on a Supervised Method

To identify the main phenotypes detected by the model, we categorized all the attractors according to the expression profiles of M1, M2 and NLC known from the literature (Table 1 and Appendix A) and the results obtained from TF estimation and gene expression profiles (Table S2c, Text S2):

- M1: IL-12, NF-κB, TNFα and STAT1 or STAT5 active;
- M2: IL-10, STAT3 or STAT6, PPARγ active;
- NLC: TGFβ, HIF1α, EGF, RAGE active;
- M0: M0 attractors + Attractors not falling in any of the above categories.

**Figure 2:**
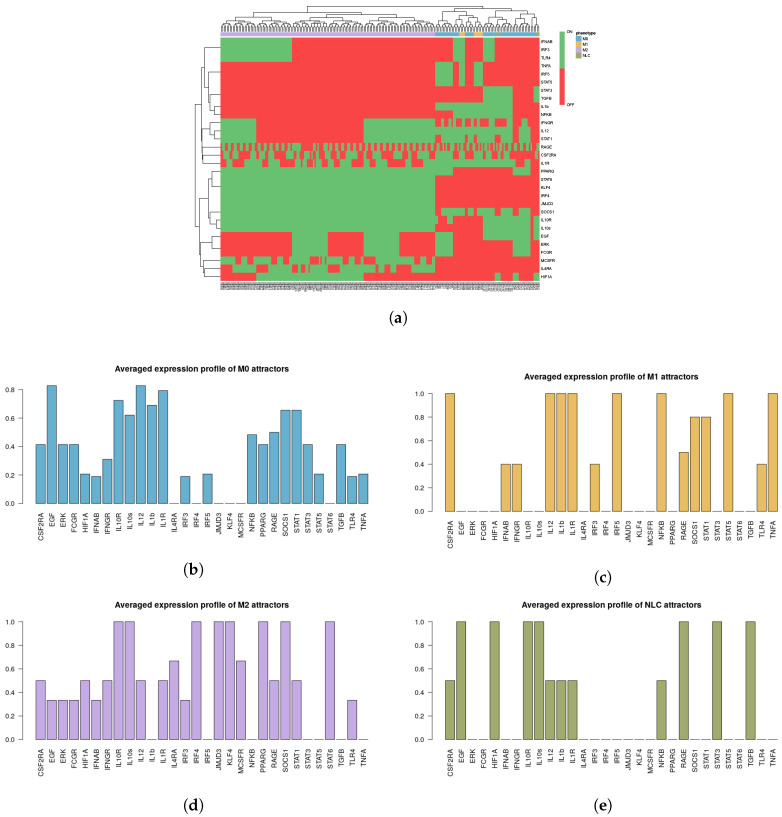
(**a**) Heatmap of 214 attractors. (**b**–**e**) Averaged attractors for each category: M0, M1, M2 and NLC.

**Figure 3:**
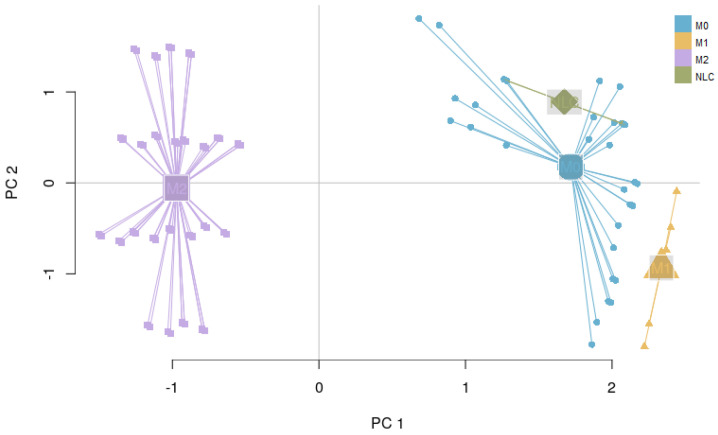
PCA of 214 attractors: M1 and M2 attractors are observed in distinct clusters, while NLC attractors appear in between the two extremes of the polarization spectrum.

It is important to note that the M1, M2 and NLC categories were considered to be mutually exclusive; therefore the rest of the attractors were categorized as M0, a separate category that includes all the attractors exhibiting characteristics of both M1 and M2 phenotypes, after removing the non-biological states (such as the all-zero attractor and an attractor with all the components in the 0 state except for RAGE = 1). To better characterize the M0 category, we removed the non-biological attractors and re-evaluated the averaged expression profile of the remaining attractors (Figure 2b). As can be seen, this category exhibits neither full M1 nor M2 characteristics, or both (high levels of EGF, IL10R, IL10, IL12, IL1β, IL1R, SOCS1, STAT1) and we believe it corresponds to a non-polarized macrophage, although more detailed characterization might be necessary [66]. Despite the existing overlap between markers of M1, M2 and NLCs, we decided to concentrate on the attractors that would have activation of markers of a specific cell type and no activation of others. Clearly other attractors exist that display a mixed phenotype but we decided to classify them separately. Interestingly, we found that most of the attractors fall into the M2 (≈67.3%) category, followed by the M1 (≈4.7%) category and NLC (≈2%) subset (Figure 2). The similarities between attractors falling in each category were estimated by calculating the Jaccard-Needham distances (dist_values ∈[0,0.5]). Considering the low values of binary distances between attractors in each category, we then calculated the average attractor states (Figure 2b–e). Importantly, we observe that these averaged attractors largely correspond to the expected expression profiles for M1, M2 and NLC defined above. A principal component analysis shows the main identified clusters of attractors corresponding to each phenotype (Figure 3). From the plot, we can easily observe that NLC attractors are not well separated from M0, which can be explained considering that a large number of attractors in our M0 category have profiles that are intermediate between M1 and M2 and NLCs are also thought to have an intermediate profile. A deeper analysis on identifying the M2 subcategories (M2a, M2b, M2c and M2d) can be found in Text S1, Figures S1 and S2.

#### 2.3.2. Interpreting Attractors Based on an Unsupervised Method

**Figure 4:**
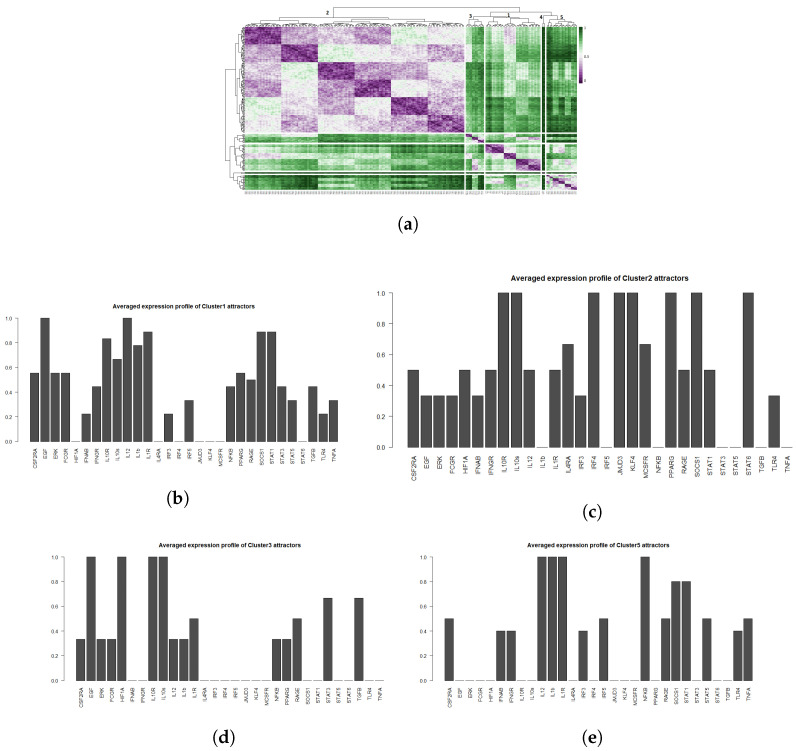
(**a**) Heatmap of Jaccard-Needham distances of 214 attractors: 5 main clusters can be observed. Cluster 4 contains the attractor with all nodes in the OFF state and was not considered for further analysis. (**b**–**e**) Averaged attractors for each cluster in (**a**).

Alongside with the supervised method, we also performed unsupervised clustering on the attractor space, in order to investigate whether the main phenotypes we expect in this system can be recovered in an unbiased way just exploring the structure of the attractors’ space. We hypothesise that the attractors corresponding to the same phenotype category will be characterized by a small binary distance and consequently will fall into the same cluster. To this end, we first estimated the similarity among the attractors by calculating the Jaccard-Needham distance [67]. We then applied hierarchical density-based clustering on the Jaccard-Needham distances (Figure 4) to identify the main attractor clusters. As can be seen from the heatmap, 5 main clusters are detected: one of them (Cluster 4) corresponds to the zero-attractors (attractor 1: all the components in OFF state, attractor 2: all the components in OFF state, except for $expr_{RAGE}=1$) and it was not considered for further analysis. A closer look at the averages of the attractors falling in each cluster highlights the detected expression profiles (Figure 4b–e). Based on the averaged expression profiles of attractors in each cluster, we observe a clear representation of M1, M2 and NLC phenotypes, respectively Cluster 5 → M1: IL-12, IL-1R, NF-κB, STAT1, TNFα highly expressed, Cluster 2 → M2: IL-10, IL-10R, JMJD3, KLF4, IRF4, PPARγ and STAT6 highly expressed, and Cluster 3 → NLC: EGF, HIF1α, RAGE, TGFβ and IL-10 highly expressed. Considering the high expression of both M1, M2 and NLC components, we attribute Cluster 1 to M0.

#### 2.3.3. Robustness of Attractor Interpretation Independent of Annotation Method

While choosing between supervised and unsupervised methods, one must consider some advantages and disadvantages. Supervised approaches can ensure a specific match between the observed attractors and prior biological knowledge of each phenotype, which can be an issue when the attractors can correspond to uncharacterised biological states and can be limited to the use of existing knowledge. On the other hand, unsupervised methods offer the simplicity of detecting the different state categories in a more unbiased way and possibly to identify unknown intermediate phenotypes in the macrophage polarization spectrum.

**Figure 5:**
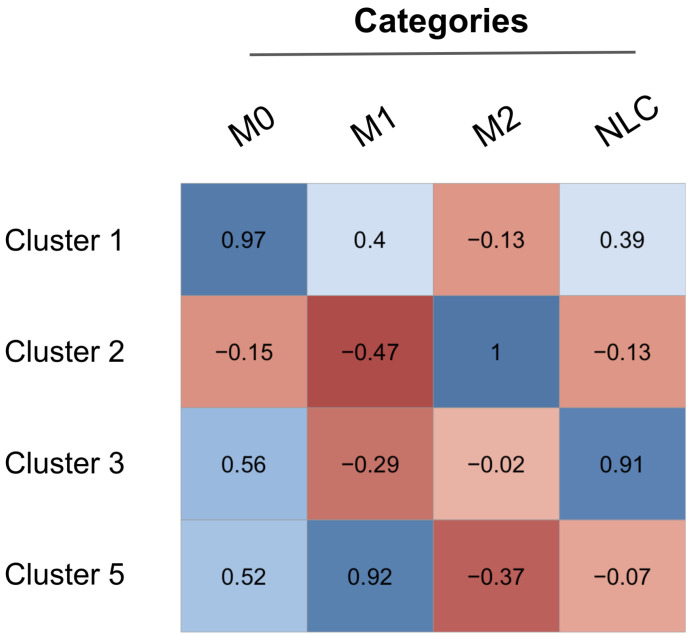
Matrix of Pearson correlation between M0, M1, M2 and NLC categories and the 4 biologically relevant clusters.

For a more quantitative comparison between the supervised and the unsupervised methods, we calculated the Pearson correlation coefficient between the averaged expression profiles obtained from each phenotype and each cluster (Figure 5). Our results show the accuracy of the unsupervised method in capturing the M1 (corr_coeff=0.92), M2 (corr_coeff=1) and NLC (corr_coeff=0.91) phenotypes, while the M0 category matches best with Cluster 1 with corr_coeff=0.97, not corresponding to any phenotype.

## 3. Model Validation through In Silico Perturbations

To validate the model, we performed several simulations mimicking specific environmental conditions consisting of M1, M2 or NLC signals only. Previous wet-lab experiments have shown that in co-cultures of monocytes and CLL cells, the CLL signal will elicit the differentiation of monocytes into NLCs. We studied the attractor space in the presence of only CLL signals (M-CSF and HMGB1) while considering all the possible combinations of intra-cellular signals. We then hypothesised that the presence of only a specific phenotype signal inducer (M1, M2 or NLC) would shift the macrophages polarization towards the corresponding phenotype and performed different simulations setting the signals favouring a certain phenotype to the ON state. Indeed, our simulations showed that the presence of specific signals (grouped as M1, M2 and NLC signals) would activate certain pathways that subsequently lead to the corresponding polarization state. Table 3 recapitulates the simulations performed by selecting only specific stimuli, the observed attractors’ categories, the expression profiles of each polarization state and the network representation of active/inactive nodes/edges under these conditions. Interestingly, we observed that while the presence of M1 and M2 signals leads to the activation of their corresponding phenotypes, NLC signals activate both M2 and NLC polarization states, which reinforces the shared pro-tumoral activity of both phenotypes in the TME.

Additionally, several experimental studies on the effects of mutants and knock-outs on macrophage polarization states have been previously published [62,68,69,70]. Here, we performed simulations of knock-outs and constitutive expressions, as summarized in Table 4. Analysing the attractors’ space, we observed a complete loss of M2 phenotype in STAT6${}^{-/-}$, IRF4-JMJD3 axis KO and a significant decrease of M2 attractors in PPAR$\gamma^{-/-}$ and IL-4R$\alpha^{-/-}$, a complete loss of M1 phenotype in IRF5${}^{-/-}$ and STAT5${}^{-/-}$, and a significant decrease in M1 attractors in STAT1${}^{-/-}$.

Finally, we performed some exploration of our model simulating the knock-out of STAT3${}^{-/-}$, in which we observed a complete loss of the NLC phenotype with an increase of M1 attractors, and knock-out of EGF${}^{-/-}$, which we predict will also lead to complete loss of the NLC attractors, whereas its constitutive activation (EGF=1) is predicted to completely eliminate M1 attractors. Interestingly, the simulated knock-down of STAT1${}^{-/-}$ is also predicted to lead to a significant loss of M1. These results show that our model recapitulates the experimental observations in mutant conditions, as well as polarization outputs in the presence of different extracellular signals. They can therefore be used to make predictions that still require experimental validation.

## 4. Discussion

The results reviewed in the previous sections highlight the various ways in which network-based dynamic models can be exploited to recapitulate the known characteristics of biological systems, as well as to predict new behaviours in specific conditions. Particularly, despite their limitations to a qualitative description, Boolean models yield a comprehensive picture of a system’s dynamics, including all the attractors of the system and the effects of mutants. Here, our main focus lies in identifying the mechanisms that trigger the formation of NLCs in Chronic Lymphocytic Leukaemia, a macrophage polarization state distinct from the ones that can be obtained with monocyte in vitro differentiation. Despite a large body of work on macrophage polarization, the phenotypic profile and formation of tumor associated macrophages have not been fully elucidated yet, due to the difficulty of isolating these cells from tumors. For this reason, we extend a previously published Boolean model of macrophage polarization [27], by including specific nodes (genes, transcription factors and receptors) that characterise the NLC profile. We then apply Boolean rules to the regulatory network to study the system’s asymptotic behaviour, when starting from all the possible initial conditions. The main macrophage polarization states (phenotypes) were matched to the attractors first by applying constraints on the value of specific network components (literature-based constraints) and subsequently using unsupervised clustering of the attractors according to their (binary) similarities. Importantly, the model results show that the attractor categories obtained by both supervised and unsupervised methods, qualitatively match the M1, M2 and NLC profiles, while highlighting specific characteristics of NLCs that distinguish them from M2 macrophages. In addition, the unsupervised method, although less accurate than the supervised approach in characterizing the phenotypes, was shown to correctly separate the phenotypic profiles in the absence of any constraint or previous knowledge. Clustering of attractors with more powerful techniques [76,77] would make the unsupervised method suitable especially in Boolean modelling of large networks for which prior biological knowledge is not available.

The ultimate test of the model presented would be to compare our in-silico signatures for the different attractors with experimental data measuring the state of each of our model components, possibly through transcriptomic or proteomic characterization of each cell type. However, the multiple levels at which the state of a component can be experimentally determined (gene expression, protein level, protein activation state) reduce our expectations for finding a clear match. Even for the well-characterised biological processes of macrophage polarization, all experimentally derived readouts of the different phenotypes come from the detection of proteins on cell membranes, leaving gaps in our understanding and justifying the need for data-driven approaches. The use of expression data to increase the model accuracy and predictive ability is becoming more and more frequent in the modelling environment and has led to the reconstruction of powerful computational methods to incorporate a wide spectrum of experimental data into the modelling language. However, in this regard, considerable inconsistencies between the literature and experimental data (like TF activity estimation or expression levels in our work) across different methods emphasize the importance of mixed approaches in modelling, especially when aiming for regulatory network inference. For example, it is widely known that IRF5 is involved in macrophage polarization toward a pro-inflammatory M1 state and IRF5 KO is associated with a reduction in expression of pro-inflammatory genes such as iNOS and TNFα, and an increase in genes associated with alternatively activated macrophages, with a loss of the M1 phenotype [70,73,78], in accordance with our KO simulations (Table 4). On the other hand, from our TF activity estimation, we observe a higher IRF5 activity in NLC compared to M1 macrophages. Further work will be devoted to investigating this inconsistency.

Here we have shown that we could construct a model of NLC formation starting from information in the literature, a pre-existing model of macrophage polarization and our own transcriptomics data for NLC produced in vitro through differentiation of monocytes in contact with cancer cells. Looking at public transcriptomics and proteomics datasets we were surprised to see relatively little overlap between the well-accepted biomarkers for M1 and M2 and the genes and proteins that are highly expressed in each specific cell type. This could be explained by the important regulatory mechanisms that lead from expression of a gene to the appearance of a protein on the cell membrane (which could be typically detected by FACS), on which most cell type descriptions in immunology are based. Nevertheless, we think that the integration of data in such a model would greatly improve both the usefulness of this model and our understanding of the different phenotypes and we will consider this approach in further work [29,79,80]. For example, Ramirez et al. [29] employed transcriptomic time courses to arrive at Boolean models of macrophages in different polarization states, showcasing the potential of integrating experimental data in our mostly literature-based approach.

On the other hand, one of the limitations of our model is the choice of a synchronous updating scheme, which prevents us from analysing the cyclic attractors which are known to change depending on the choice of update rules. We think that more biological knowledge about the processes described by this model will be needed to design a better and more appropriate update scheme to then explore the attractor space more deeply.

We are also aware that NLCs could represent an artificial phenotype which we observe in the specific in vitro conditions of our experiment and that in patients there could be oscillations spanning all the macrophage phenotypes mentioned. However, since M1 and M2 can clearly be related to the presence of specific stimulatory signals, we think the presence of cancer cells should lead to at least a predominance (over cells in a population or over time) of the NLC state. NLCs have a very specific behaviour in CLL, sending anti-apoptotic signals to the cancer cells, with a very important clinical impact. In the future, we plan to consider more advanced updating schemes that will allow us to explore complex attractors (as mentioned above) which are almost certainly also relevant and could better reflect the nature of NLCs.

Taken together, our model can describe macrophage polarization in different environments and mutant conditions. The inflammatory and cancer environments are characterized by a complex combination of stimuli, which drive the polarization process of monocytes towards specific macrophage phenotypes. In our network, we include the most significant pro- and anti-inflammatory signals, as well as important cytokines that are involved in NLC polarization, such as CSF-1 (M-CSF in our model) and HMGB1. Despite the specific characteristics of the tumor micro-environments in solid cancers compared to the in vitro model considered here, we believe that common polarization pathways are also involved in the formation of tumor associated macrophages (TAMs) in solid tumors, which have so far been modeled with a stronger emphasis on the inter-cellular aspects than on the molecular details [81,82,83]. Further work will be needed to establish whether our model can be useful more generally in different cellular environments.

Despite the limitations of our model and of the in vitro system described, we can allow ourselves to hypothesize about the possible relevance of this model in explaining the mechanistic aspects of formation of TAMs in solid tumors and to suggest potential strategies for hindering their formation. With the advent of immunotherapy, the low response to immune checkpoint blockers that target CD8${}^{+}$ T cells for reactivation in a large proportion of patients remains a serious issue. TAMs are thought to play an important role in this, with the observation that a high macrophage infiltration in tumors in specific indications or patients is often predictive of weaker CD8 infiltration and lower response to current immunotherapies. In particular, over 20% of lung cancer patients carry EGFR mutations that produce a constitutive activation of the EGF receptor [84]. We predict an effect of EGF knock-outs and constitutive activations on the proportion of attractors corresponding to M1 and NLC (see Table 4). Patients carrying EGFR mutations show a reduced response to immunotherapy [85], suggesting that further exploration of EGF’s role in the formation of TAM might be of clinical relevance. Overall, we hope that our model will encourage new empirical investigations on the complex nature of cell-cell interactions in the TME and the role of TAMs in cancer prognosis and treatment.

## 5. Methods

### 5.1. Boolean Model Implementation

Here we describe how we implemented a Boolean model starting from the regulatory network described above. In the Boolean model each component (gene/mRNA, protein, chemokine) is associated with a discrete (binary) variable, representing its concentration, activity or expression [86,87,88]. Time is considered to be implicit and the future states of each component were determined by the current states of their regulators, given by a Boolean function of $m_{i}=1,2,\ldots ,N$ regulators of component $X_{i}$. Each Boolean function represents the regulatory relationships between the components and is expressed via Boolean operators AND, OR and NOT. The state of the system at each time point is given by a binary vector, whose *i*th element represents the state of the component $X_{i}$ [24,26]. The set of all possible states and their transitions can be represented by a state transition graph, in which the nodes are the system’s states (represented as binary vectors) and the directed edges are the transitions between them. The exponential function between the number of components and the state space size makes the graphical representation possible for only small networks. In Boolean models time is discrete and implicit: starting from an initial state, the system will follow a trajectory of states and, because of the finite state space, it reaches an attractor (stable states or limit cycles). To evaluate the state of each node at each timestep, two main updating methods have been proposed [22,89]:

- *synchronous updating method*: at each time step, all the nodes are updated simultaneously, assuming that all the interactions in the system require the same time to occur. Importantly, the state space is characterized by non-overlapping basins of attractions.
- *asynchronous updating method*: at each time step, the updated nodes are chosen randomly (General Asynchronous, Random Asynchronous) or according to their *characteristic updating time*, while the system’s state will be characterized by overlapping basins of attractions.

It is important to note that fixed point attractors are time invariant, i.e., do not depend on the updating method. Choosing the synchronous update method we obtain all transitions between them and consider the final attractors. Our network is composed of N=40 components (excluding the 3 output nodes which are added for clarity in Figure 1) and has $2^{40}$ possible states. The model was implemented using the BoolNet [69] R package [90].

### 5.2. Calculating the Attractor Similarity Matrix

In this section we give the mathematical background of the similarity measures used for estimating the similarity between different attractors, which we then clustered according to these similarity indexes. Here we hypothesise that the attractors representing a specific phenotype will be characterized by a small distance index (i.e., higher similarity).

Given Ω a space of binary *N*-dimensional vectors *Z* defined as

(1) $$Z=\left(z_{1},z_{2},...,z_{N}\right),z_{i}=\left\{0,1\right\},\forall i\in \left\{1,2,...N\right\}$$

we define $\bar{Z}=1-Z$ to be the complement of the binary vector *Z*. For each set of binary vectors $Z_{1},Z_{2}\in \Omega$ let $S_{ij}$ be the number of occurrences of matches, with $i\in Z_{1}$ and $j\in Z_{2}$ being in the corresponding positions. In this way $S_{11}\left(Z_{1},Z_{2}\right)=Z_{1}\cdot Z_{2}$ and $S_{00}\left(Z_{1},Z_{2}\right)=\bar{Z_{1}}\cdot \bar{Z_{2}}$.

Based on $S_{ij}$, different measures exist, to calculate the similarity/dissimilarity between two binary vectors [67]. For our purpose, we calculated the *Jaccard-Needham* measures, defined as follows:

(2) $$S\left(Z_{1},Z_{2}\right)=\frac{S_{11}}{S_{11}+S_{10}+S_{01}}\;\left(similarity\right)\;D\left(Z_{1},Z_{2}\right)=\frac{S_{10}+S_{01}}{S_{11}+S_{10}+S_{01}}\;\left(dissimilarity\right)$$

### 5.3. Calculating the Transcription Factor Activities

Since gene expression of a TF is not representative of its activity level and many of the nodes in our network are TFs, we sought to estimate activity of these components using bioinformatic methods that consider the expression of a TF’s targets taken from a dataset in combination with a regulatory network database. We extracted gene expression data from different public sources. Microarray data used in this publication were downloaded from the NCBI repository Gene Expression Omnibus (GEO) database. M1 and M2 Macrophages microarray data accession number is GSE5099 [51,52]. Our previously published NLC microarray dataset can be found under accession number GSE87813 and was processed as described in [55]. Raw microarray datasets were then normalized using the RMA (Robust Multi-arrays Average) normalization method and batch corrected. Transcription factors activities were estimated using the Dorothea R package and ISMARA. Dorothea is a TF-regulon interaction database giving each interaction a confidence level. Here, levels of confidence of interactions from A to E were taken into account. The VIPER algorithm was used to estimate TF activities based on Dorothea interactions and our expression data [53]. ISMARA is a web-based tool to identify the key TFs and miRNAs driving expression/chromatin changes and to predict activities of the regulators across the samples, their genome-wide targets, enriched gene categories among the targets, and direct interactions between the regulators [54]. For both methods, the comparison between TF activities across the phoneotypes (M1, M2, and NLC) is performed using the rank method. The complete analysis of TF estimation from both packages, as well as TF activity comparison between phenotypes, can be accessed in Material, Tables S2a–d and S3a–d.

## 6. Conclusions

We present a Boolean model of macrophage polarization in the presence of cancer cells. We showcase two alternative ways to annotate the attractors into previously identified macrophage types, which include M1, M2, and NLC, the specific TAMs encountered in CLL. We validate our model by simulating knockouts that have been experimentally performed in mouse models and make new predictions regarding the importance of STAT and EGF in regulating the production of TAMs, with possible implications for their control in solid tumors.

## Figures and Tables

**Table 1:** The main characteristics of M1, M2 and NLC/TAM phenotypes according to (i) activators, (ii) secreted cytokines or expressed genes, and (iii) functions in tumoral environments (see Appendix A). In blue we indicate the components used in our polarization network and in bold the components used for the signature for each phenotype.

	M1	M2	NLC/TAM
**Activated by**	IFNγ LPS GM-CSF IL-1β	Immune complexes (IC) IL-4 IL-13 IL-10	Immune complexes (IC) IL-4 IL-13 IL-10 TGFβ CSF-1 HMGB1
**Secrete**	Th1 inducing cytokines TNFα IL-12 IL-18 IFNα/β IL-1β IL-6	IL-10 TGFβ EGF VEGF Polyamine	IL-10 TGFβ EGF VEGF TNFSF13/TNFSF13B (BAFF/APRIL) chemokines: CXCL12, CXCL13 Polyamine
**Function**	**Anti-tumoral activity:** releasing nitric oxide (NO); presenting tumor antigens to CD4+ Th1 cells; driving the activity of cytotoxic CD8+ T cells at the tumor site	**Anti-infammatory processes:** Th2 responses(M2a); Down-regulation of immune response (M2b); matrix deposition and tissue remodelling (M2c)	**Promote tumor growth:** secretion of soluble immuno- suppressive agents; expression of contact-dependent immuno-suppressive receptors (PD-L1, B7-H4) leading to enhancing CD8${}^{+}$ T cell infiltration high levels of HIF1 and HIF2 which leads to expression of genes associated with pro-tumoral activity
**References**	[1,36,37,38,39,40]	[1,2,3,38]	[12,13,17,37,38,41,42,43,44]

**Table 2:** Boolean rules of the 30 intra-cellular nodes of the macrophage polarization network (Table S1b–c).

Node	Boolean Function	References
IFNGR	IFNG or IFNAB and not (SOCS1)	[27,59]
CSF2RA	GMCSF	[27]
IL1R	IL1 or IL1b	[27]
TLR4	LPS and not (FCGR)	[27]
FCGR	IC and (LPS or IL1)	[27]
IL4RA	IL4 and IL13	[1,2,7]
IL10R	IL10 or IL10s (secreted)	[27]
MCSFR	MCSF(also known as CSF-1)	[7,12,44,55,60]
STAT1	IFNGR or STAT1 and not (STAT6)	[1,13,45,61]
STAT5	CSF2RA and not (STAT3 or IRF4)	[27]
NFKB	(STAT1 or TNFA or TLR4 or IL1R)	[7,27,42,62]
	and not (STAT6 or FCGR or PPARG or KLF4)	
PPARG	IL4RA or MCSFR or ERK and not (STAT6)	[2,37,62,63]
STAT6	IL4RA or MCSFR	[18,27,37,45,55]
JMJD3	IL4RA or MCSFR	[18,27,37,55]
STAT3	(IL10R or EGF or STAT3) and not (FCGR or PPARG)	[27,62,64,65]
IRF3	TLR4	[27]
ERK	FCGR	[27]
KLF4	STAT6	[27]
SOCS1	STAT6 or STAT1	[2,27,59]
IRF4	JMJD3	[27]
IRF5	STAT5 and not (IRF4)	[1,2,57]
IL1b	NFKB or TNFA	[36,38]
IFNAB	IRF3	[27]
EGF	ERK or STAT3	[41,43,44]
IL12	STAT1 or STAT5 or NFKB	[27]
IL10s	(PPARG or STAT3) and not (IRF5 or TNFA)	[7,39,42,45]
TNFA	IRF5 and not (IL10s)	[39,42]
TGFB	STAT3 and (not TNFA)	[7,36,38,41,42,43,44]
HIF1A	(STAT3 or IL10s) and (not STAT1)	[38,40,43,44]
RAGE	HMGB1	[37,42,63]

**Table 3:** Simulations of environmental signals consisting of M1-, M2- and NLC-inducing signals only.

Simulations	Attractors	Network Representation
M1 stimuli ON: IFNγ, GM-CSF, IL-1, LPS. Attractor category: 18 M0, 5 M1	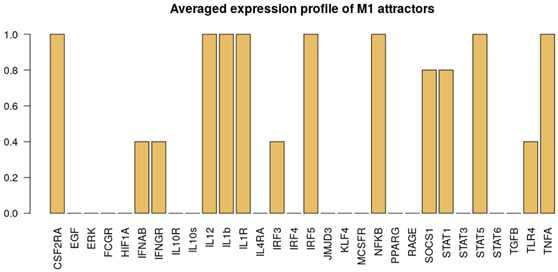	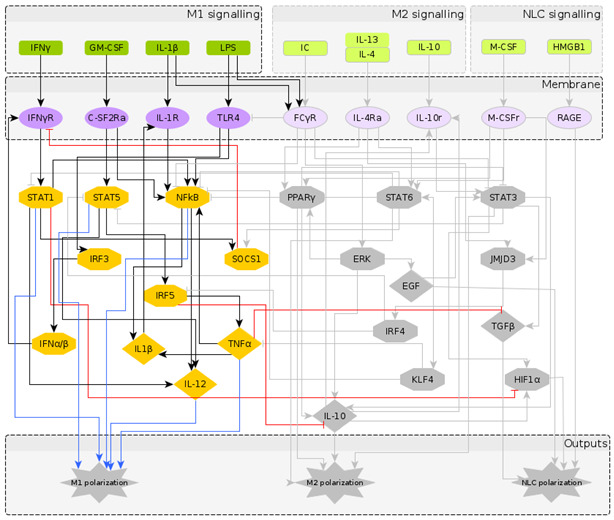
M2 stimuli ON: IC, IL-4, IL-13, IL-10. Attractor category: 6 M0, 1 M2	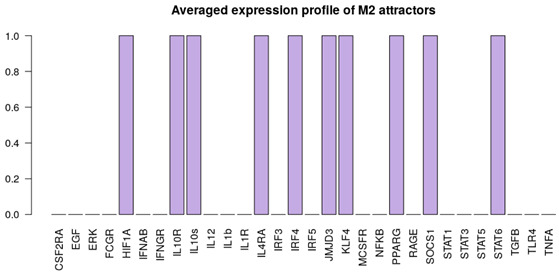	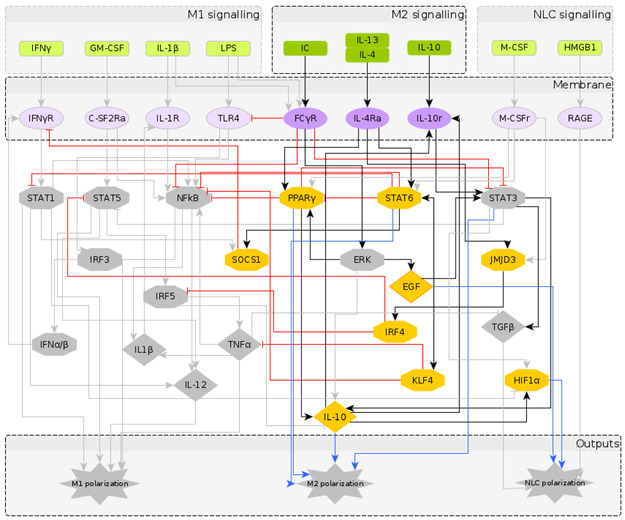
NLC stimuli ON: M-CSF, HMGB1. Attractor category: 4 M0, 2 M2, 4 NLC	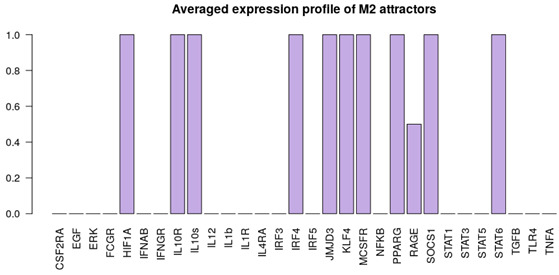	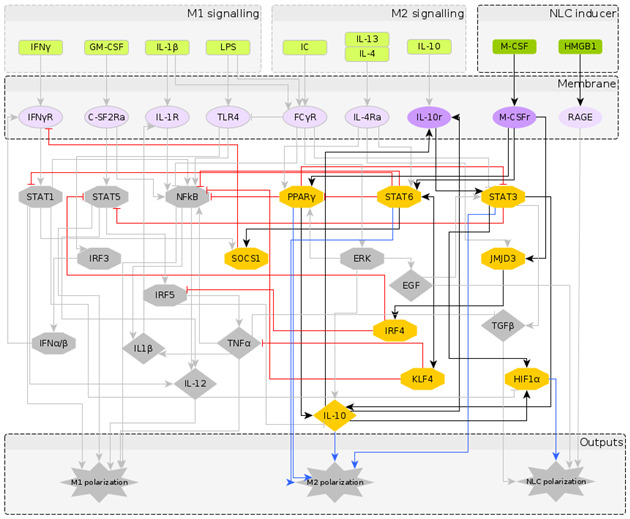
	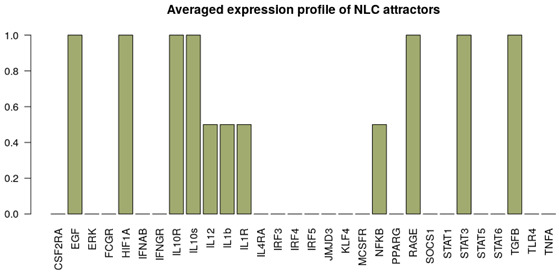	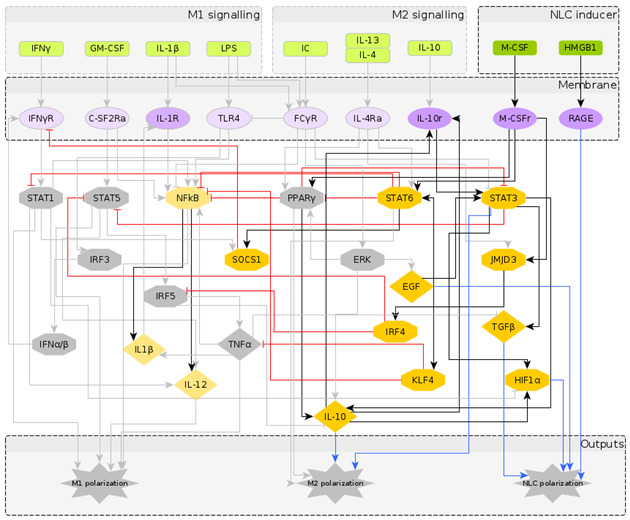

**Table 4:** In-Silico experiments with knock-outs and constitutive activations compared to experimental results [70].

Knock-Out	Expected Effect on Polarization	Model Results
STAT6	Complete knock-out Loss of M2 [71]	Complete loss of M2 M1 and NLC attractors not affected
PPARγ	Conditional knock-out Loss of M2 [62]	Decrease number of M2 attractors M1 and NLC attractors not affected
IL-4Rα	Conditional knock-out Loss of M2 [72]	Decrease number of M2 attractors M1 and NLC attractors not affected
IRF5	Complete knock-out Loss of M1 [73]	Complete loss of M1 M2 and NLC attractors not affected
STAT5	Complete knock-out Loss of M1 [74]	Complete loss of M1 M2 and NLC attractors not affected
IRF4 - JMJD3 axis	Complete knock-out Loss of M2 [75]	Complete loss of M2 M1 and NLC attractors not affected
**Predictions**		
**Node Perturbed**	**Mutant**	**Model Result**
STAT3	Knock-out	Complete loss of NLC Increase of M1 attractors M2 attractors not affected
EGF	Knock-out	Complete loss of NLC M1 and M2 attractors not affected
STAT1	Knock-out	Significant loss of M1 M2 and NLC attractors not affected
EGF	Constitutive Activation	Complete loss of M1 M2 and NLC attractors not affected

## Supplementary Materials

The following are available online at https://www.mdpi.com/2072-6694/12/12/3664/s1, Text S1: M2 macrophage sub-categories. Text S2: Expression profiles of Monocyte and M1, M2 and NLC macrophages. Text S3: List of Supplementary Tables. Figure S1: M2 subcategories. Figure S2: Averaged expressions of M2 subcategories. Figure S3: Principal Component Analysis for 5 samples of monocytes, 3 samples of M1, M2 and 19 samples of NLC. Figure S4: Gene expression profiles for monocyte, M1, M2 and NLC for (a) cytokines secreted, (b) transcription factors and (c) receptors. Figure S5: Transcription factor activity for the TF nodes of regulatory network of macrophage polarization. Figure S6: Comparison between gene expression and TF activity estimated with Dorothea/VIPER and ISMARA packages for 19 NLC microRNA samples. It can be seen that the two packages give significantly different results on TF activity, and also considerable difference with gene expression analysis. All distributions (gene expression, TF activities) were independently normalized between 0 and 1 from the results obtained for the NLC phenotype only by subtracting the minimum value min(x) and dividing by distribution range max(x) - min(x). Even if a direct comparison between ISMARA and Dorothea/VIPER TF activity estimated values is not permitted, a lack of correlation is clearly observable. Figure S7: Heatmap of gene expression of 5 monocyte samples, 3 M1 and M2 samples and 19 NLC samples. Table S1a: Macrophage regulatory network extension: added nodes. Table S1b: The main similarities and differences from macrophage regulatory network extension. Table S1c: Boolean rules for the added nodes in the macrophage polarization network. Table S2a: dorothea_results.tsv. Table S2b: Differential activity (Dorothea, M1 > M2 and NLC). Table S2c: Differential activity (Dorothea, M2 > M1 and NLC). Table S2d: Differential activity (Dorothea, NLC > M1 and M2). Table S3a: ismara_results.tsv. Table S3b: Differential activity ranking (ISMARA, M1 > M2 and NLC). Table S3c: Differential activity ranking (ISMARA, M2 > M1 and NLC). Table S3d: Differential activity ranking (ISMARA, NLC > M1 and M2).

## Appendix A

### Appendix A.1. M1 Pathway

The M1-like pro-inflammatory polarization state is applied to pro-inflammatory macrophages and can be obtained upon stimulation of those cells with IFNγ or LPS which cause release of Th1-inducing cytokines including tumor necrosis factor α (TNFα), IL-12, IL-6, IL-1β, IL-18 and IFNα/β [1,36,37]. The M1 macrophages metabolism rely on oxidative glycolysis [91] and intrinsically their polarization is linked with activation of STAT1, IRF5 and NF-κB [92]. M1-like macrophages are linked in fighting bacterial infections and intracellular pathogens. Additionally they show potent anti-tumoral activity which manifests mainly through: (i) release of large amount of nitric oxide (NO), which in turn is able to kill the cancer cells as a result of DNA damage, disruption of mitochondrial activity and limitation of iron availability, and (ii) presentation of tumor antigens to CD4${}^{+}$ Th1 cells and driving the activity of cytotoxic CD8${}^{+}$ T cells at the tumor site [38].

### Appendix A.2. M2 Pathway

M2-like macrophages include a wide variety of phenotypes involved in resolving of the inflammation. The M2 activation can be induced by stimulation with IL-4, IL-13, immune complexes and IL-10. The anti-inflammatory and regenerative activity of M2 macrophages come from abundant release of IL-10, TGF-β, VEGF and EGF [1,38]. M2 macrophages depend strongly on oxidative phosphorylation [91] and the main TFs driving their polarization-state are: STAT6, PPARγ/δ, IRF4, JMJD3 [92]. Depending on the anti-inflammatory processes M2-like macrophages are involved in, they manifest diverse phenotypes including: M2a-Th2 responses and killing and encapsulation of parasites, M2b—immunoregulation, M2c—matrix deposition and tissue remodeling [1,2,3].

Tumor-associated macrophages belong to the group of cells that arise upon the contact with cancer cells and tumor microenvironment (TME). They can show characteristics of both M1 and M2 state, nevertheless upon prolonged presence in the TME the M2 characteristic becomes prevalent. TAMs influence the properties and dynamics of TME, although the precise factors that promote TAM activation have yet to be elucidated, as each TME is characterized by unique physical and chemical conditions [13,38]. However, certain common features may be identified. For example, CSF1, IL-10 and TGF-β released from tumor cells and Treg cells, are powerful promoters of TAM polarization, which in turn support tumor progression by various mechanisms, such as: (i) secretion of soluble immunosuppressive agents (IL-10, TGF-β, IL-1β), (ii) expression of Immune Checkpoint Inhibitors (PD-L1, B7-H4), and (iii) high levels of hypoxia-inducible factor 1 and 2 (HIF1, HIF2) which leads to expression of genes associated with pro-tumoral activity [12,13,38,93].

In the context of Chronic Lymphocytic Leukaemia (CLL) it has been proposed that Nurse-like cells (NLC), which are specific form of TAMs identified in this malignancy, are polarized in response to CSF-1 and HMGB1 proteins released by CLL cancer cells. In turn NLCs can stimulate and protect CLL cells by antigen presentation which stimulates BCR signaling, and also by both direct contact through membrane proteins and release of soluble factors including: [37]

- **membrane proteins:** CD2 (interacts with LFA-3 expressed on CLL cells [55]), CD31 (ligand of CD38 expressed on CLL cells), BAFF, APRIL (both BAFF and APRIL can be also released as soluble factors) [37,55]
- **soluble factors:** BDNF [94], WNT5A [95], CXCL12, CXCL13, IL6/8, IL-10 [37].
