# Supplementary material for: Insights on TAM Formation from a Boolean Model of Macrophage Polarization Based on In Vitro Studies
Source: Cancers (Basel). 2020 Dec 7;12(12):3664. doi: 10.3390/cancers12123664 (PMC7762229; doi:10.3390/cancers12123664)
Supplement: Supplementary file 1 [file cancers-12-03664-s001.zip › cancers-980693-supplementary/Supplementary Material.pdf]

Supplementary Material

Insights on TAM Formation from a Boolean Model of Macrophage Polarization based on *in-vitro* Studies

Malvina Marku, Nina Verstraete, Flavien Raynal, Miguel Madrid-Mencía, Marcin Domagala, Jean-Jacques Fournié, Loïc Ysebaert, Mary Poupot and Vera Pancaldi

Supplementary Text S1

M2 macrophage sub-categories

Here, we include the results from a deeper analysis on the M2 category of attractors. From the results obtained from the Boolean model of macrophage polarization, which captures the formation of M1, M2 and NLC phenotypes in the presence of a combination of extracellular signals, we aimed to detect the M2 sub-categories, named M2a, M2b, M2c and M2d by applying clustering algorithms on the M2 category of attractors. The full description of M2 sub-categories can be found in (Palma et al 2018, Martinez et al 2014, Gordon 2010) and the references therein. Our analysis shows the presence of 4 main clusters of attractors in the M2 category (Figure S1). For a better understanding of the expression levels of each component, we then averaged across the attractors in each cluster. The barplots of these averaged expression profiles are represented in Figure S2 (a)-(d). Next, we referred to the Palma et al model (Palma et al 2018), where a description of 3 out of 4 M2 sub-categories are given (M2a, M2b and M2c), as follows:

- M2a: all of PPARγ, STAT6, JMJD3 and IL-10 are active;
- M2b: ERK and IL-10 are active;
- M2c: STAT3 and IL-10 are active.

By comparing the averaged expressions given by the model and the criteria for M2a, M2b and M2c, we can associate M2a to either clusters 1, 2, 3, M2b to cluster 4, but M2c does not fit to any of these clusters. We believe that additional constraints must be applied in the simulations in order to observe clearly the M2 sub-categories.

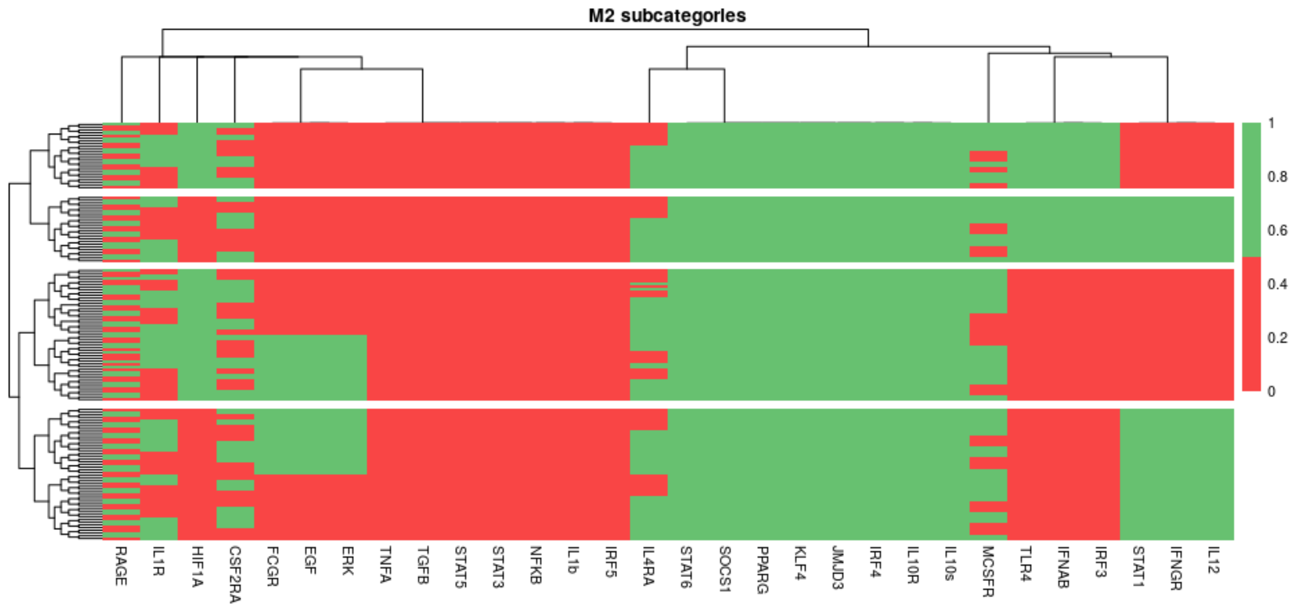

Figure S1: M2 subcategories

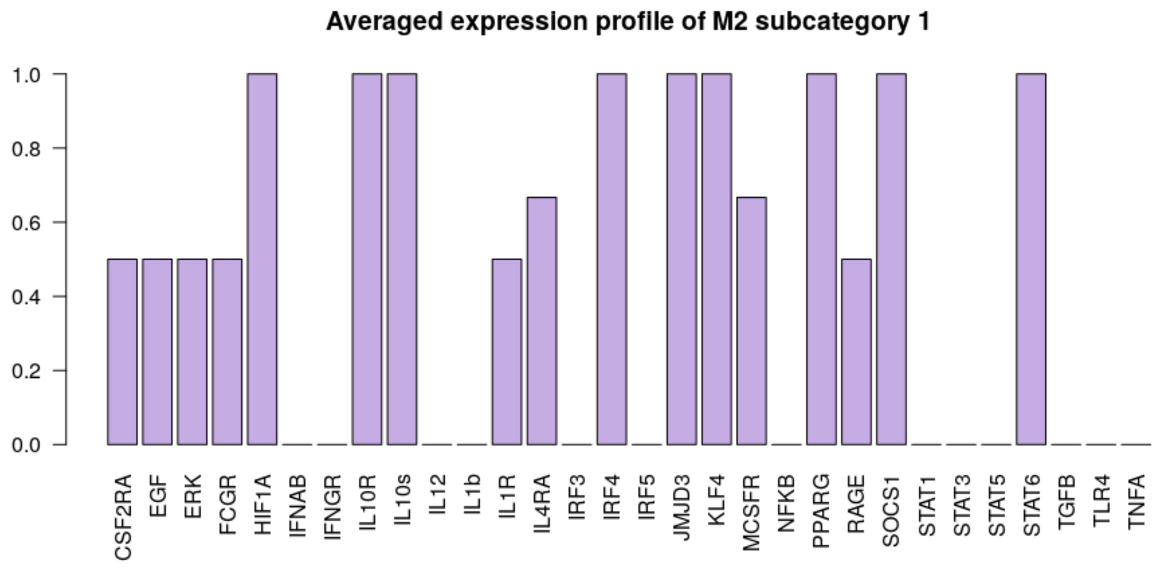

(a)

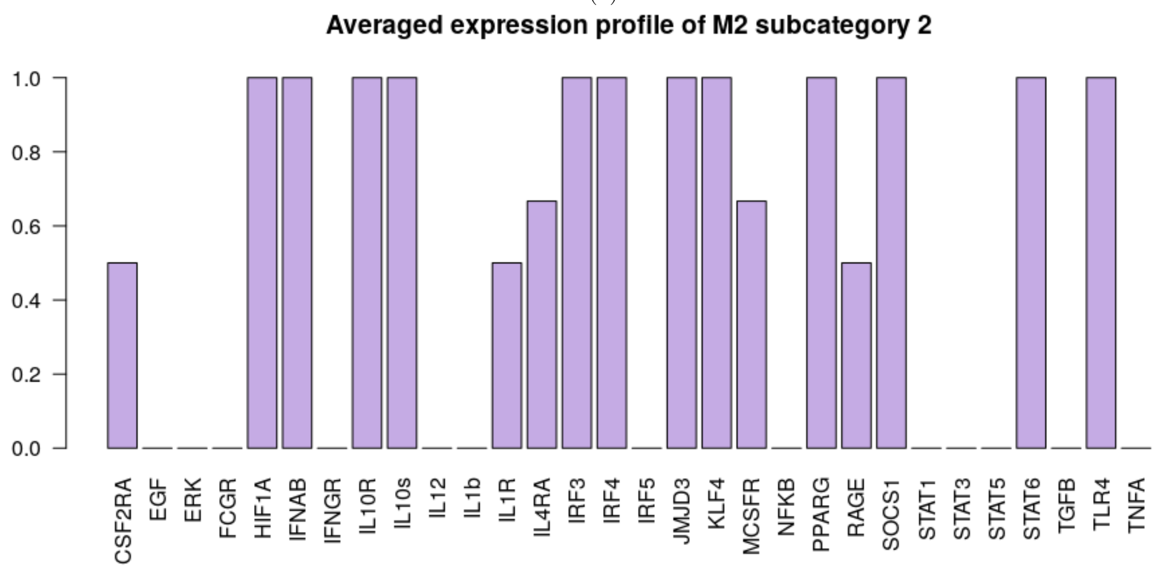

(b)

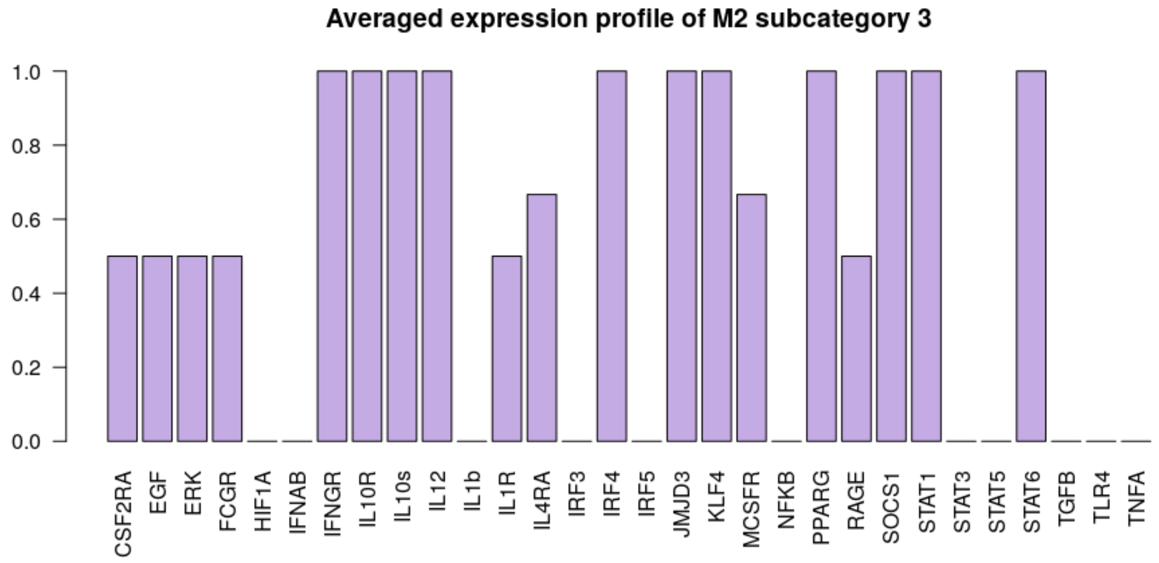

(c)

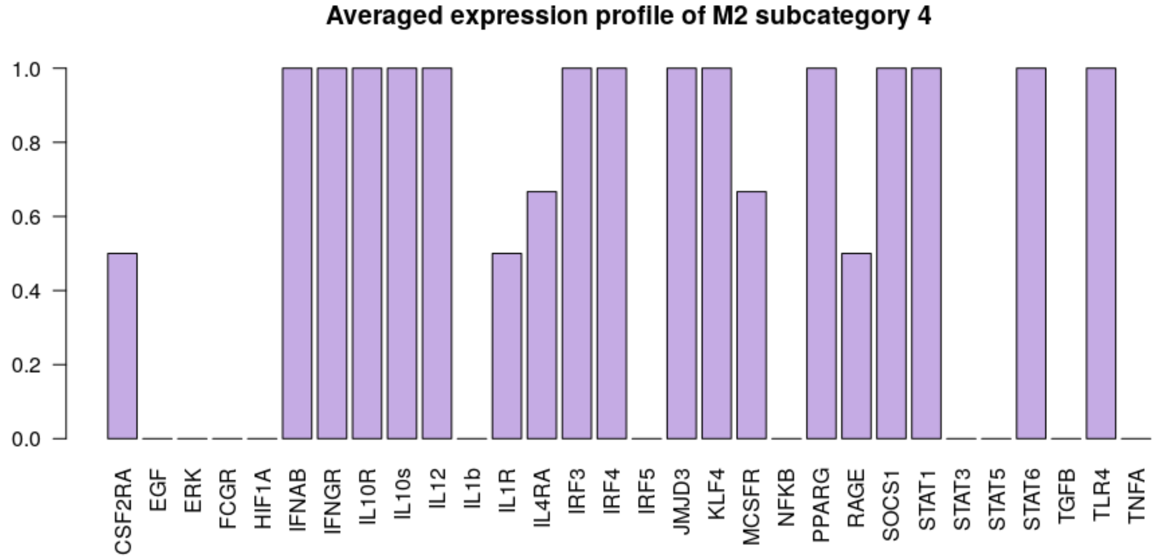

(d)

Figure S2: Averaged expressions of M2 subcategories.

Supplementary Text S2

Expression profiles of Monocyte and M1, M2 and NLC macrophages

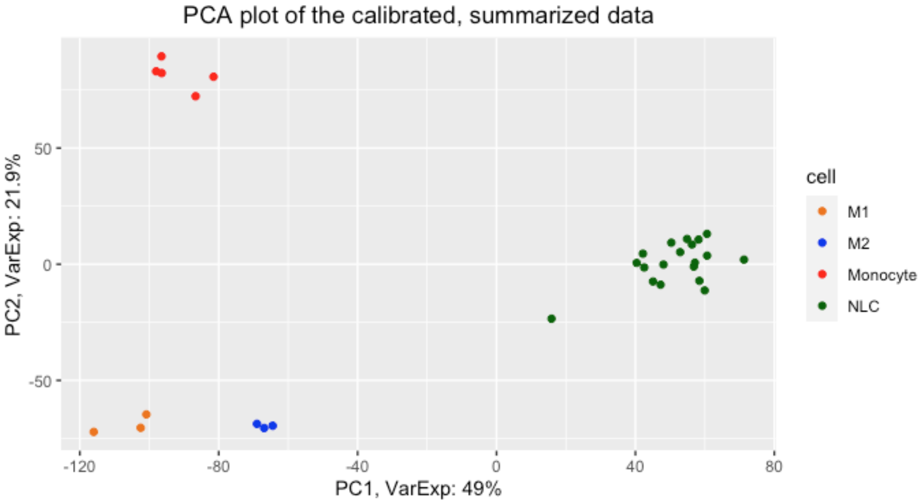

Figure S3: Principal Component Analysis for 5 samples of monocytes, 3 samples of M1, M2 and 19 samples of NLC.

Figure S4 (a) – (c) represent the expression levels of cytokines, transcription factors and receptors of the regulatory network of macrophage polarization, represented in Figure 1, for monocytes, and M1, M2 and NLC macrophages obtained from 5 monocyte, 3 M1 and M2 (Martinez et al 2006, Solinas et al 2010), and 19 NLC samples (Boissard et al 2017).

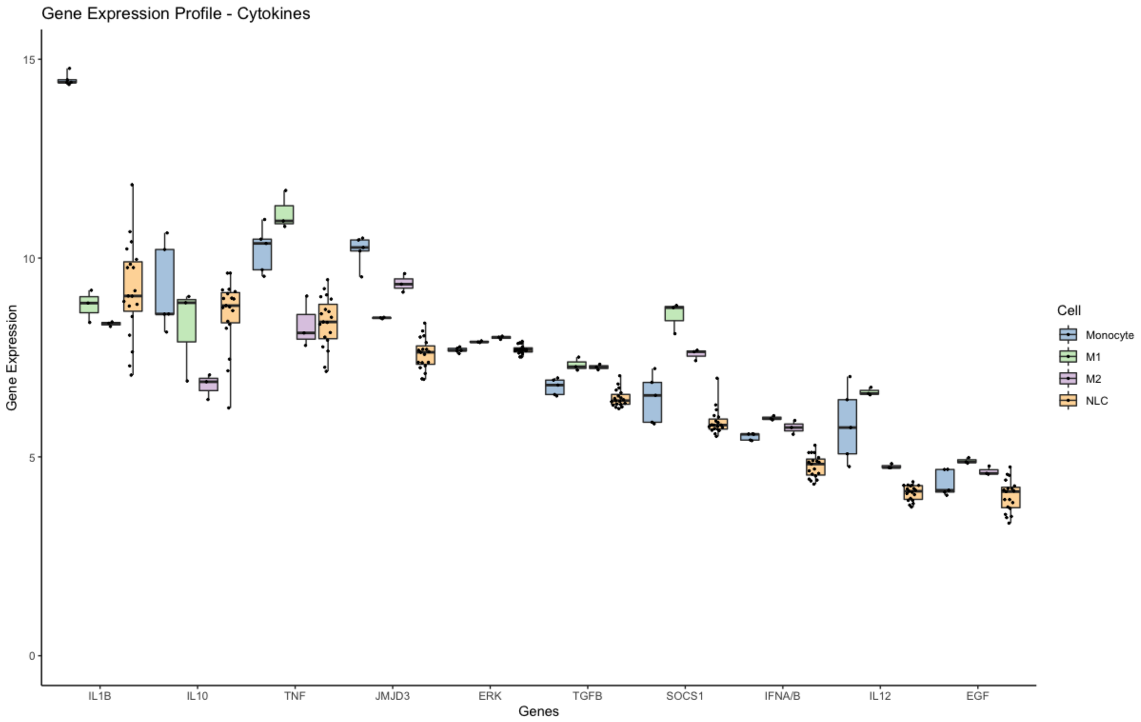

(a)

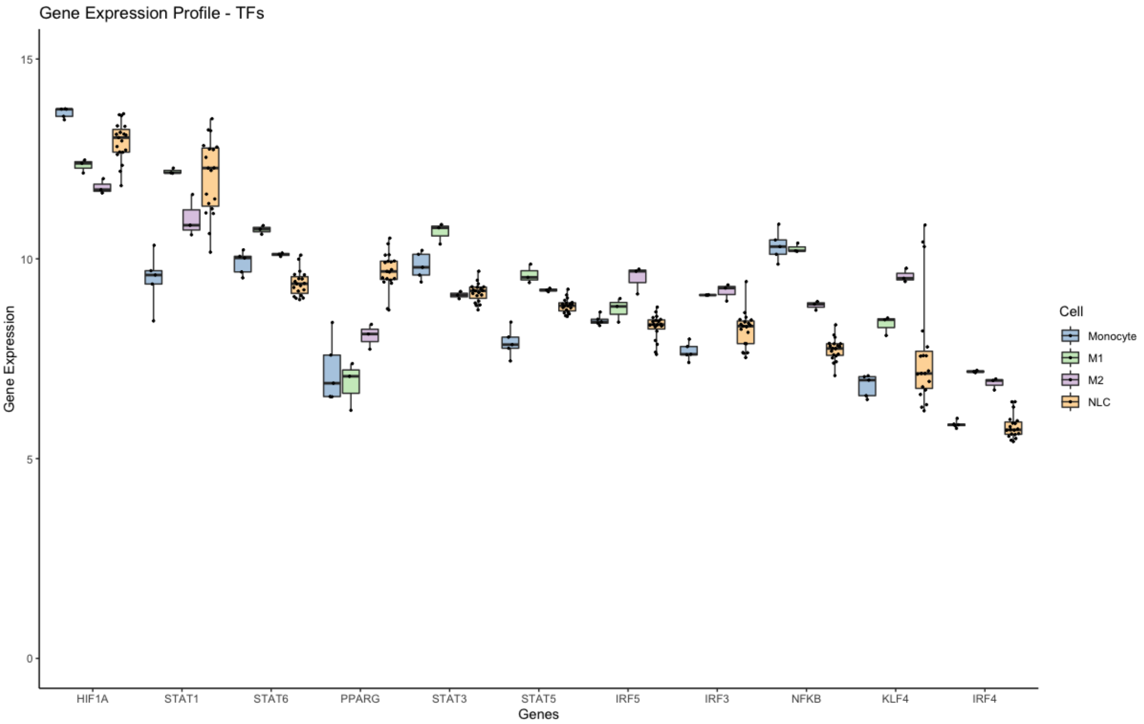

(b)

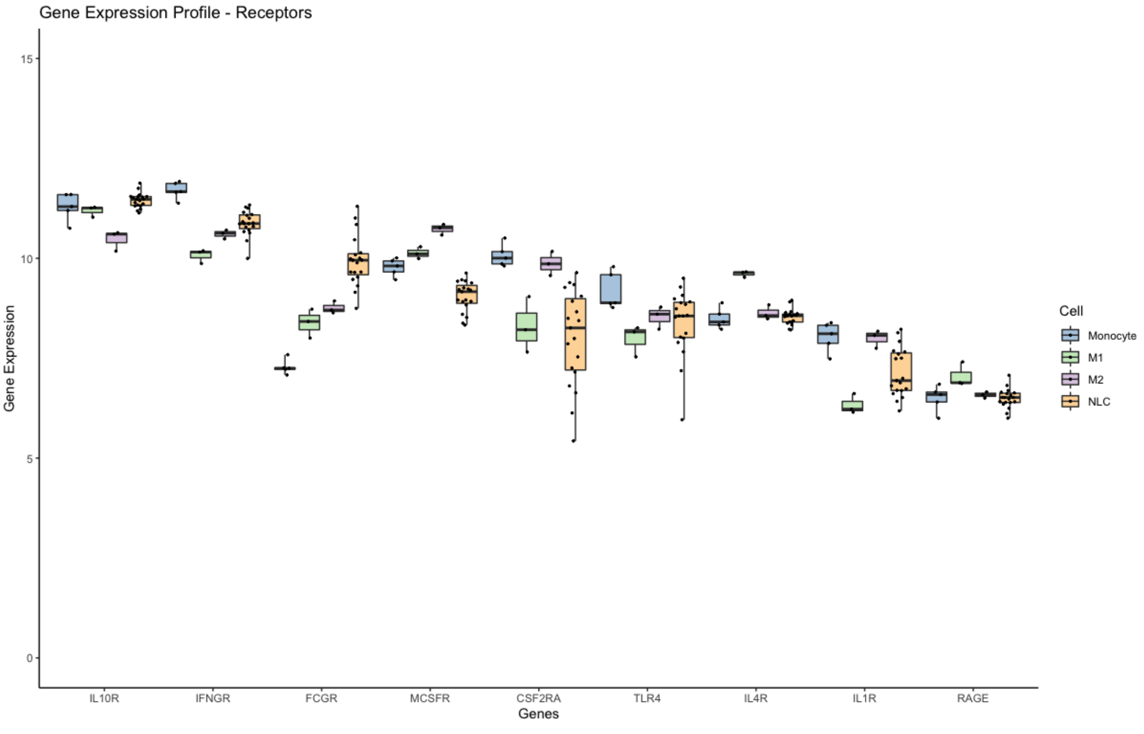

(c)

Figure S4: Gene expression profiles for monocyte, M1, M2 and NLC for (a) cytokines secreted, (b) transcription factors and (c) receptors.

TF activity is not simply dependent on the expression of the mRNA corresponding to the TF protein. First the mRNA needs to be translated to a protein, a highly regulated process; second the functioning of TFs often relies on post-translational modifications such as phosphorylation which cannot be captured by the translated protein levels. For this reason, we resorted to 2 separate methods that infer TF activity from expression data. Dorothea is a TF-regulon interaction database giving each interaction a confidence level. Here, levels of confidence of interactions from A to E were taken into account. The VIPER algorithm was used to estimate TF activities based on Dorothea interactions and our expression data (Holland et al, Garcia-Alonso et al 2019). ISMARA is a web-based tool to identify the key TFs and miRNAs driving

expression/chromatin changes and to predict activities of the regulators across the samples, their genome-wide targets, enriched gene categories among the targets, and direct interactions between the regulators (Balwierz et al 2014). For both methods, the comparison between TF activities across the phonotypes (M1, M2 and NLC) is performed using the rank method. The complete analysis of TF estimation from both packages, as well as TF activity comparison between phenotypes can be accessed in supplementary tables listed List of Supplementary Tables.
Using Dorothea package, we estimated the transcription factor (TF) activity of each phenotype. The results of TF activities for the TF nodes in the regulatory network of Figure 1 are shown in Figure S5.

We were able to use the TF activity estimates to check whether our proposed added TFs in the model (HIF1a and IRF5) could be shown to be comparatively more active in NLCs from Dorothea (rank method). The TF activity analysis shows that among the 10 added nodes, the two TFs HIF1A and IRF5 are more active in NLC than in M1 and M2 (see the TF activity heatmap in the Figure S5 below), supporting their *potential* role in NLC polarization.

It also supports the TFs chosen to be included in each phenotype's signature:

- M1 signature: NFKB, STAT1 and STAT5 are more active in M1 compared to M2 and NLC
- M2 signature: STAT6 and PPARG are more active in M2 compared to M1 and NLC. STAT3 does not follow the same pattern, and this discrepancy shows the limits of using TF activity level as a way to determine "marker" proteins or key regulators.
- NLC signature: HIF1A is more active in NLC compared to M1 and M2.

We doubt that the most active TFs in each phenotype can be directly considered as TF markers that will define the phenotypes' signatures. However, we believe that differential activity analysis among the different phenotypes (see heatmap below (Figure S5) and the Supplementary Tables S2b-d) can help us confirm the appropriateness of the TF markers chosen for the signatures, as well as some added nodes (HIF1a and IRF5).

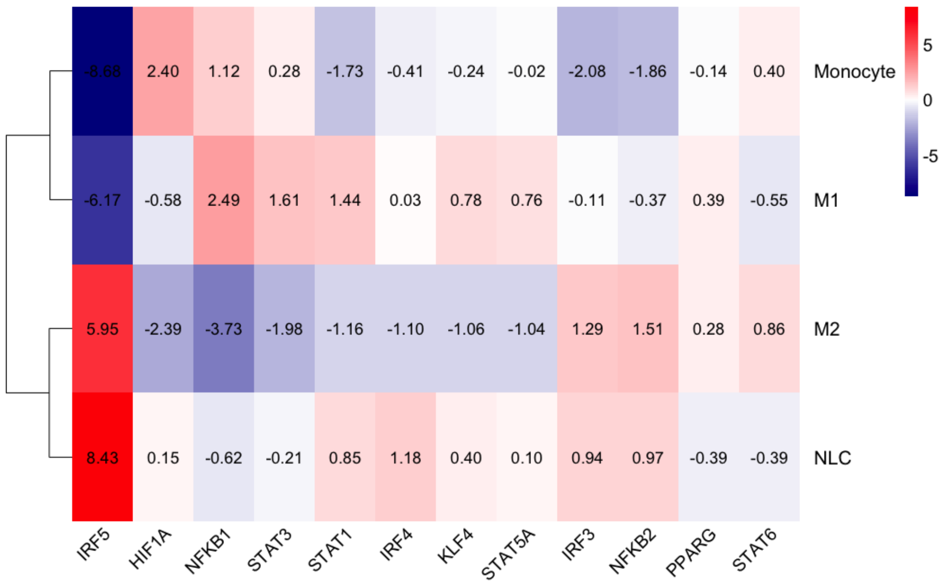

Figure S5: Transcription factor activity for the TF nodes of regulatory network of macrophage polarization.

Figure S6 shows the gene expression level, and the corresponding TF activity estimated with Dorothea and ISMARA for the TF nodes of the macrophage polarization network of Figure 1. We observe that gene expression levels do not directly correlate with TF activity, reinforcing the importance of including this analysis in our model.

Figure S7 shows a comparison between gene expression profiles of the monocyte, M1, M2 and NLC samples used in this work.

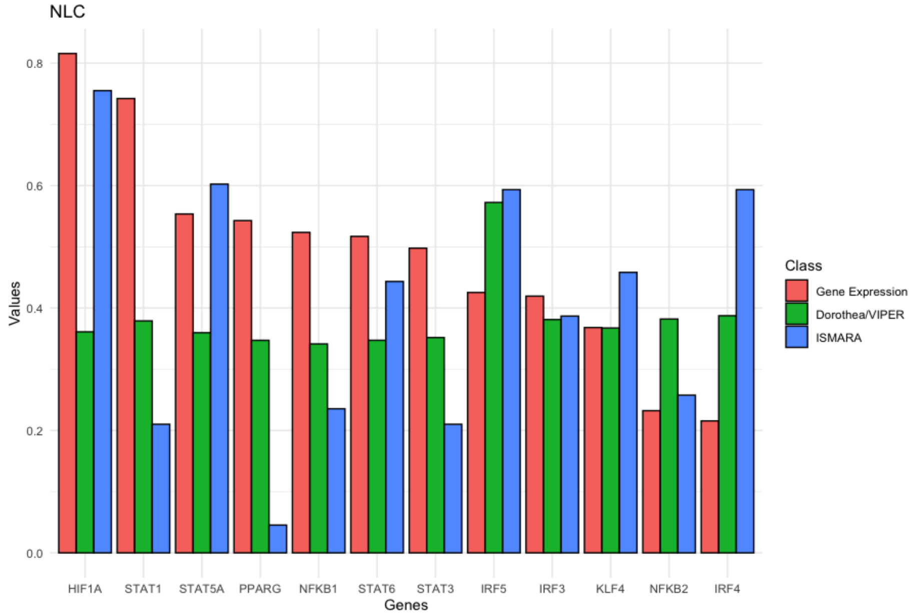

Figure S6: Comparison between gene expression and TF activity estimated with Dorothea/VIPER and ISMARA packages for 19 NLC microRNA samples. It can be seen that the two packages give significantly different results on TF activity, and also considerable difference with gene expression analysis. All distributions (gene expression, TF activities) were independently normalized between 0 and 1 from the results obtained for the NLC phenotype only by subtracting the minimum value min(x) and dividing by distribution range max(x) - min(x). Even if a direct comparison between ISMARA and Dorothea/VIPER TF activity estimated values is not permitted, a lack of correlation is clearly observable.

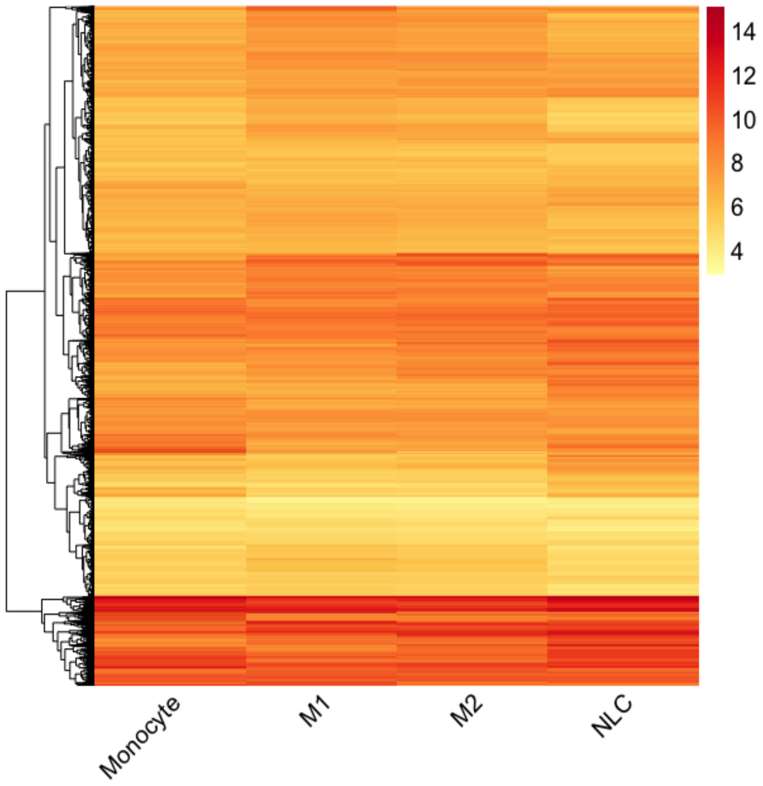

Figure S7: Heatmap of gene expression of 5 monocyte samples, 3 M1 and M2 samples and 19 NLC samples.

Supplementary Text S3

List of Supplementary Tables

The supplementary tables used in this work can be accessed in the Supplementary Material, in which we show the results obtained from calculating the transcription factor activity with Dorothea/VIPER (Holland et al, Garcia-Alonso et al 2019) and ISMARA (Balwierz et al 2014) packages. Additionally, we searched for the TFs whose activities are higher in M1 vs M2 and NLC, M2 vs M1 and NLC vs M1 and M2, sorting them by descending differential activity. This procedure could be done directly based on TF estimated activities only on Dorothea results. On the other hand, ISMARA activity estimations could not be compared across phenotypes (different samples), therefore in this case, we compared the TFs according to their ranks in each phenotype, also sorted by descending differential rankings.

The tables in Supplementary Material are as follows:

- Table S2a: `dorothea_results.tsv`
- Table S2b: Differential activity (Dorothea, M1 > M2 and NLC)
- Table S2c: Differential activity (Dorothea, M2 > M1 and NLC)
- Table S2d: Differential activity (Dorothea, NLC > M1 and M2)
- Table S3a: `ismara_results.tsv`
- Table S3b: Differential activity ranking (ISMARA, M1 > M2 and NLC)
- Table S3c: Differential activity ranking (ISMARA, M2 > M1 and NLC)
- Table S3d: Differential activity ranking (ISMARA, NLC > M1 and M2)

Table S2a displays TF names and TF activities in M1, M2 and NLC as estimated by Dorothea (rank method). For comparison purposes, i.e. in order to have only positive values, we have also added columns displaying scaled activities corresponding to the activities shifted by the minimum activity found among M1, M2 and NLC (i.e. activity → activity - Min(actM1, actM2, actNLC)).

Tables S2b-d display the same data as in table S2a, but filtered for TFs whose activity is higher in M1 vs. M2 and NLC (Table S2b), higher in M2 vs. M1 and NLC (Table S2c) or higher in NLC vs. M1 and M2 (Table S2d). An additional column displays the difference between scaled activities between the indicated phenotypes, as a way to measure the TF's differential activity across these phenotypes. Finally, the TFs lists were sorted from the highest differential activity to the lowest.

Similarly, Table S3a displays TF names and TF activities as estimated in M1, M2 and NLC by ISMARA. As mentioned above, we did not consider ISMARA estimations to be comparable across samples/phenotypes, so in this case, we have added columns displaying the ranking of activities values in each phenotype (the lowest the rank the minimum the activity and the highest rank the highest the activity; and if some activity values are equal, their ranking position is calculated using the average).

Tables S3b-d display the same data as in Table S3a but filtered for TFs whose activity ranking is higher in M1 vs. M2 and NLC (Table S3b), higher in M2 vs. M1 and NLC (Table S3c) or higher in NLC vs. M1 and M2 (Table S2d). An additional column displays the difference between the activities rankings between the indicated phenotypes, as a measure to evaluate the TF's differential activity ranking across these phenotypes. The TFs lists were sorted from the highest differential ranking to the lowest.

Supplementary Table S1a

Macrophage regulatory network extension: added nodes

Node	Type	Inference based on	Implications	Reference
**IL13**	Signal	Literature	Binds to IL4 (direct interaction)<br>IL4 – IL13 form a dimer	Mueller et al, 2002<br>Foey, 2014
**HMGB1**	Signal	Literature	Released by CLL cells can stimulate NLC differentiation through activation of the receptor for advanced glycation end-product (RAGE)- toll like receptor 9 (TLR9) pathway.	Hao et al, 2012<br>ten Hacken et al, 2016<br>Jia et al, 2014<br>Choi et al, 2014
**M-CSF**	Signal	Literature	Promotes cancer cell proliferation, invasion and formation of metastases	Li et al, 2020<br>Ries et al, 2014
**M-CSFR**	Receptor	Literature	Promotes cancer cell proliferation, invasion and formation of metastases<br>Receptor for M-CSF<br>Required for NLC survival	Li et al, 2020<br>Ries et al, 2014
**RAGE**	Receptor	Literature	Establishes HMGB1 - NLC interaction<br>**Chosen as key marker for NLC**	ten Hacken et al, 2016<br>Jia et al, 2014
**TNFa**	Cytokine	Literature + confirmed by gene expression profile	Pro-inflammatory cytokine<br>Induces cell death of certain tumour cell lines<br>Activates NF-kB and IL-1b<br><br>**Chosen as key marker for M1**	Kratochvill et al, 2015<br>Gkikas et al, 2018
**TGFb**	Growth factor	Literature	TAMs release growth factors such as TGF-β, EGF, which can promote angiogenesis in many tumours, immuno-suppression;<br>Promotes tumour-associated macrophage polarization;<br>**Chosen as key marker for NLC**	Hao et al, 2012<br>Li et al, 2020<br>Guttman et al, 2016
**HIF1a**	TF	Literature + confirmed by TF estimation	TAMs are found to accumulate in hypoxic regions of tumours, controlled by HIF1/2<br>HIF1A has been shown to be closely associated with cancer development<br>and progression<br>Regulates CXCL12<br>**Chosen as key marker for NLC**	Hao et al, 2012<br>Gao et al, 2014<br>Schioppa et al, 2003<br>Solinas et al, 2009
**EGF**	Growth factor	Literature	TAMs release growth factors such as TGF-β, EGF which can promote angiogenesis in many tumours<br>Up-regulates the activity of STAT3<br>**Chosen as key marker for NLC**	Hao et al, 2012<br>Li et al, 2020<br>Guttman et al, 2016
**IRF5**	TF	Literature + confirmed by TF estimation	Down-regulates quantity of IL10 by repression<br>Activates M1-polarization<br>Up-regulates quantity of TNFa by expression	Martinez et al, 2014<br>Murray et al, 2014<br>Signor

Supplementary Table S1b

The main similarities and differences from macrophage regulatory network extension

Palma et al model	Model extension, Marku et al
**Macrophage polarization states**	
• Classically activated macrophages M1<br>• Alternatively activated macrophages M2<br>• M2a, M2b, M2c	• Classically activated macrophages M1<br>• Alternatively activated macrophages M2<br>• Tumour Associated Macrophages in Chronic Lymphocytic Leukaemia (Nurse Like Cells, NLC)
**Regulatory network**	
30 nodes<br>• 7 extracellular signals (inputs)<br>• 27 intracellular components (cytokines, TFs)<br>• Inference: literature	40 nodes<br>• 10 extracellular signals (inputs)<br>• 30 intracellular components (cytokines, TFs)<br>• Added components (Table S2):<br>Signals: HMGB1, M-CSF, IL13<br>Receptors: M-CSFR, RAGE<br>Intracellular: TGFb, TNFa, IRF5, HIF1a, EGF<br>• Inference: literature, interaction databases
**Boolean model**	
• Synchronous updating scheme<br>• Boolean rules inference based on literature	• Synchronous updating scheme<br>• Boolean rules inference based on literature
**Attractors space, categorization across phenotypes**	
• 1040 fixed point attractors<br>• 78% do not fall in any category (not included in further analysis)<br>• The remaining (228 attractors) categorize according to the frequency: M2a, M2c, M1, M2b	• 1384 fixed point attractors<br>• 214 attractors (inputs excluded)<br>• Categorization in M1, M2, NLC<br>1. Supervised (based on literature, TF estimation, gene expression profile);<br>2. Unsupervised (based on similarity estimation across attractors)<br>• Attractor frequency across phenotypes: M2, M1, NLC

Supplementary Table S1c

Boolean rules for the added nodes in the macrophage polarization network

Boolean rule		Description
IFNgR	IFNG | IFNAB & !(SOCS1)	IFNg binds to its receptor. SOCS1 is key inhibitor or IFNgR (Palma et al 2018, Satou et al 2012)
IL4Ra	IL4 & IL13	IL4/IL13 dimer binds to IL4R receptor (Van Dyken et al 2013)
MCSFR	MCSF	MCSF bind to its receptor (Duluk et al 2007)
RAGE	HMGB1	HMGB1 binds to its receptor (ten Hacken et al 2016)
PPARg	IL4RA | MCSF | ERK & !(STAT6)	PPARg is activated in response to IL4/IL13 and ERK. MCSFR activates IRF4 through PPARg (Palma et al 2018, Gordon et al 2010, Nemenoff et al 2008, Kim et al 2018, Ivashkiv 2013, Lawrence et al 2011)
STAT6	IL4RA | MCSFR	STAT6 is activated by IL4 receptor and M-CSFR (Palma et al 2018, Gordon 2003, Lawrence et al 2011, Ivashkiv 2013, Chen et al 2018)
JMJD3	IL4RA | MCSFR	JMJD3 is activated by IL4 receptor and MCSFR (Palma et al 2018, Ivashkiv 2013)
STAT3	(IL10R | EGF | STAT3) & !(FCGR | PPARG)	STAT3 is activated in response to IL10 and EGF, and inhibited by PPARg and FCgR (Zhong et al 1994, Palma et al 2018, Henson et al 2006, Ivashkiv 2013, Jin 2020)
STAT1	IFNGR | STAT1 & !(STAT6)	IFNgR activates JAK/STAT1 pathway, while STAT6 inhibits STAT1 (Palma et al 2018, Ohmori et al 1997)
NFkB	(STAT1 | TNFA | TLR4 | IL1R) & !(STAT6 | FCGR | PPARG | KLF4)	NFkB is activated by IL1/LPS signalling pathways and inhibited by M2-related pathways (Palma et al 2018, Fang et al 2018, Liu et al 2017, Lawrence et al 2011)
SOCS1	STAT6 | STAT1	SOCS1 is activated by STAT6 and STAT1 (Palma et al 2018, Penrose et al 2020, Satou et al 2012)
IL1b	NFKB | TNFA	IL1b is activated by NFkB and TNFa (Palma et al 2018, Lawrence et al 2011, Álvarez et al 2013)
IL10s	(PPARG | STAT3) & !(IRF5 | TNFA)	PPARg and STAT3 downstream genes lead to IL-10 production while pro-inflammatory cytokines inhibit its expression (Palma et al 2018, Gordon 2003, Lawrence et al 2011, Foey 2014, Gordon et al 2010, Krausgruber et al 2011, Donnelly et al 1995, Jin 2020)
TNFa	IRF5 & !(IL10s)	IRF5 activates TNFa secretion while IL-10 inhibits cytokine production of TNFa (Hoepel et al 2019, Fiorentino et al 1991, Clarke et al 1998)
TGFb	STAT3 & (!TNFA)	STAT3 activates TGFb through JAK/STAT3 pathway, while TNFa inhibits TGFb (Jin 2020, Szondy et al 2017)
HIF1a	(STAT3 | IL10s) & (!STAT1)	STAT3 and IL10 activate HIF1a expression through positive regulation of angiogenesis. STAT1 represses HIF1 transcription (Jin 2020, Wu et al 2010, Hiroi et al 2009)
EGF	ERK | STAT3	ERK mediates downstream regulation of EGF. JAK/STATe pathways regulates pro-tumoral genes (Li et al 2008, Jin 2020)
IRF5	STAT5 & !(IRF4)	STAT5 activates IRF5 while IRF4 inhibits IRF5 expression (Martinez et al 2014, Thompson et al 2018, Ni et al 2016)

References

Álvarez, S. and Muñoz-Fernández, M.Á.. TNF-A may mediate inflammasome activation in the absence of bacterial infection in more than one way. *PLoS One* **2013**, *8*(8), p.e71477.

Balwierz, P.J.; Pachkov, M.; Arnold, P.; Gruber, A.J.; Zavolan, M.; van Nimwegen, E. ISMARA: automated modeling of genomic signals as a democracy of regulatory motifs. *Genome research* **2014**, *24*, 869–884.

Boissard, F.; Tosolini, M.; Ligat, L.; Quillet-Mary, A.; Lopez, F.; Fournié, J.J.; Ysebaert, L.; Poupot, M. Nurse-like cells promote CLL survival through LFA-3/CD2 interactions. *Oncotarget* **2017**, *8*, p.52225.

Chen, H.M., van der Touw, W., Wang, Y.S., Kang, K., Mai, S., Zhang, J., Alsina-Beauchamp, D., Duty, J.A., Mungamuri, S.K., Zhang, B. and Moran, T.. Blocking immunoinhibitory receptor LILRB2 reprograms tumor-associated myeloid cells and promotes antitumor immunity. *The Journal of clinical investigation* **2018**, *128*(12), p.5647-5662.

Clarke, C.J., Hales, A., Hunt, A. and Foxwell, B.M. IL-10-mediated suppression of TNF-α production is independent of its ability to inhibit NFκB activity. *European journal of immunology* **1998**, *28*(5), p.1719-1726.

Donnelly, R.P., Freeman, S.L. and Hayes, M.P. Inhibition of IL-10 expression by IFN-gamma up-regulates transcription of TNF-alpha in human monocytes. *The Journal of Immunology* **1995**, *155*(3), p.1420-1427.

Duluc, D., Delneste, Y., Tan, F., Moles, M.P., Grimaud, L., Lenoir, J., Preisser, L., Anegon, I., Catala, L., Ifrah, N. and Descamps, P. Tumor-associated leukemia inhibitory factor and IL-6 skew monocyte differentiation into tumor-associated macrophage-like cells. *Blood* **2007**, *110*(13), p.4319-4330.

Fang, J., Muto, T., Kleppe, M., Bolanos, L.C., Hueneman, K.M., Walker, C.S., Sampson, L., Wellendorf, A.M., Chetal, K., Choi, K. and Salomonis, N. TRAF6 mediates basal activation of NF-κB necessary for hematopoietic stem cell homeostasis. *Cell reports* **2018**, *22*(5), p.1250-1262.

Fiorentino, D.F., Zlotnik, A., Mosmann, T.R., Howard, M. and O'garra, A. IL-10 inhibits cytokine production by activated macrophages. *The Journal of immunology* **1991**, *147*(11), p.3815-3822.

Foey, A.D. Macrophages — Masters of Immune Activation, Suppression and Deviation. *Immune Response Activation* **2014**.

Foey, A.D. Macrophages—masters of immune activation, suppression and deviation. *Immune Response Activation* **2014**, p.121-149.

Gao, B.; Wang, Y.; Qiu, Y.; Xia, W.; Meng, X.; Lu, M.; Zhang, W.; Qiao, X.; Zhang, Y.; Xue, D. Bioinformatics screening regarding herbal components that targetedly regulate the function of tumour-associated macrophages. *Oncology Reports* **2014**, *32*, p.309–317.

Garcia-Alonso, L.; Holland, C.H.; Ibrahim, M.M.; Turei, D.; Saez-Rodriguez, J. Benchmark and integration of resources for the estimation of human transcription factor activities. *Genome research* **2019**, *29*, 1363–1375.

Gkikas, I.; Palikaras, K.; Tavernarakis, N. The role of mitophagy in innate immunity. *Frontiers in immunology* **2018**, *9*, p.1283.

Gordon, S. Alternative activation of macrophages. *Nature reviews immunology* **2003**, *3*(1), p.23-35.

Gordon, S. and Martinez, F.O. Alternative activation of macrophages: mechanism and functions. *Immunity* **2010**, *32*(5), p.593-604.

Guttman, O.; C. Lewis, E. M2-like macrophages and tumor-associated macrophages: overlapping and distinguishing properties en route to a safe therapeutic potential. I*ntegrative Cancer Science and Therapeutics* **2016**, *3*, p.554–561.

Hao, N.B.; Lü, M.H.; Fan, Y.H.; Cao, Y.L.; Zhang, Z.R.; Yang, S.M. Review Article Macrophages in Tumor Microenvironments and the Progression of Tumors. *Clinical and Developmental Immunology* **2012**, *11*.

Henson, E.S. and Gibson, S.B. Surviving cell death through epidermal growth factor (EGF) signal transduction pathways: implications for cancer therapy. *Cellular signalling* **2006**, *18*(12), p.2089-2097.

Hiroi, M., Mori, K., Sakaeda, Y., Shimada, J. and Ohmori, Y. STAT1 represses hypoxia-inducible factor-1-mediated transcription. *Biochemical and biophysical research communications* **2009**, *387*(4), p.806-810.

Hoepel, W., Newling, M., Vogelpoel, L.T., Sritharan, L., Hansen, I.S., Kapsenberg, M.L., Baeten, D.L., Everts, B. and den Dunnen, J. FcγR-TLR cross-talk enhances TNF production by human monocyte-derived DCs

via IRF5-dependent gene transcription and glycolytic reprogramming. *Frontiers in Immunology* **2019**, *10*, p.739.

Holland, C.H.; Valdeolivas, A.; Saez-Rodriguez, J. TF activity inference from bulk transcriptomic data with DoRothEA as regulon resource.

Ivashkiv, L.B. Epigenetic regulation of macrophage polarization and function. *Trends in immunology* **2013**, *34*(5), p.216-223.

Jia, L.; Clear, A.; Liu, F.T.; Matthews, J.; Uddin, N.; McCarthy, A.; Hoxha, E.; Durance, C.; Iqbal, S. and Gribben, J.G. Extracellular HMGB1 promotes differentiation of nurse-like cells in chronic lymphocytic leukemia. *Blood, The Journal of the American Society of Hematology* **2014**, *123(11)*, p.1709-1719.

Jin, W. Role of JAK/STAT3 signaling in the regulation of metastasis, the transition of cancer stem cells, and chemoresistance of cancer by epithelial–mesenchymal transition. *Cells* **2020**, *9*(1), p.217.

Kim, M.J., Lee, Y.J., Yoon, Y.S., Kim, M., Choi, J.H., Kim, H.S. and Kang, J.L. Apoptotic cells trigger the ABCA1/STAT6 pathway leading to PPAR-γ expression and activation in macrophages. *Journal of Leukocyte Biology* **2018**, *103*(5), p.885-895.

Kratochvill, F.; Neale, G.; Haverkamp, J.M.; Van de Velde, L.A.; Smith, A.M.; Kawauchi, D.; McEvoy, J.; Roussel, M.F.; Dyer, M.A.; Qualls, J.E.; others. TNF counterbalances the emergence of M2 tumor macrophages. *Cell reports* **2015**, *12*, p.1902–1914.

Krausgruber, T., Blazek, K., Smallie, T., Alzabin, S., Lockstone, H., Sahgal, N., Hussell, T., Feldmann, M. and Udalova, I.A. IRF5 promotes inflammatory macrophage polarization and TH 1-TH 17 responses. *Nature immunology* **2011**, *12*(3), p.231-238.

Lawrence, T. and Natoli, G. Transcriptional regulation of macrophage polarization: enabling diversity with identity. *Nature reviews immunology* **2011**, *11*(11), p.750-761.

Li, X., Huang, Y., Jiang, J. and Frank, S.J. ERK-dependent threonine phosphorylation of EGF receptor modulates receptor downregulation and signaling. *Cellular signalling* **2008**, *20*(11), p.2145-2155.

Li, Y.; You, M.J.; Yang, Y.; Hu, D.; Tian, C. The Role of Tumor-Associated Macrophages in Leukemia. *Acta haematologica* **2020**, *143*, p.112–117.

Liu, T., Zhang, L., Joo, D. and Sun, S.C. NF-κB signaling in inflammation. *Signal transduction and targeted therapy* **2017**, *2*(1), p.1-9.

Martinez, F.O.; Gordon, S. The M1 and M2 paradigm of macrophage activation: Time for reassessment. *F1000Prime Reports* **2014**, *6*, p.1–13

Mueller, T.D.; Zhang, J.L.; Sebald, W.; Duschl, A. Structure, binding, and antagonists in the IL-4/IL-13 receptor system. *Biochimica et Biophysica Acta (BBA)-Molecular Cell Research* **2002**, *1592(3)*, p.237-250.

Murray, P.J.; Allen, J.E.; Biswas, S.K.; Fisher, E.A.; Gilroy, D.W.; Goerdt, S.; Gordon, S.; Hamilton, J.A.; Ivashkiv, L.B.; Lawrence, T.; Locati, M.; Mantovani, A.; Martinez, F.O.; Mege, J.L.; Mosser, D.M.; Natoli, G.; Saeij, J.P.; Schultze, J.L.; Shirey, K.A.; Sica, A.; Suttles, J.; Udalova, I.; VanGinderachter, J.A.; Vogel, S.N.; Wynn, T.A. Macrophage Activation and Polarization: Nomenclature and Experimental Guidelines. *Immunity* **2014**, *41*, p.14–20

Nemenoff, R.A., Weiser-Evans, M. and Winn, R.A. Activation and molecular targets of peroxisome proliferator-activated receptor-γ ligands in lung cancer. *Ppar Research* **2008**, *2008*.

Ni, Y., Zhuge, F., Nagashimada, M. and Ota, T. Novel action of carotenoids on non-alcoholic fatty liver disease: macrophage polarization and liver homeostasis. *Nutrients* **2016**, *8*(7), p.391.

Ohmori, Y. and Hamilton, T.A. IL-4-induced STAT6 suppresses IFN-gamma-stimulated STAT1-dependent transcription in mouse macrophages. *The Journal of immunology* **1997**, *159*(11), p.5474-5482.

Palma, A., Jarrah, A.S., Tieri, P., Cesareni, G. and Castiglione, F. Gene regulatory network modeling of macrophage differentiation corroborates the continuum hypothesis of polarization states. *Frontiers in physiology* **2018**, *9*, p.1659.

Penrose, H.M., Katsurada, A., Miyata, K., Urushihara, M. and Satou, R. STAT1 regulates interferon-γ-induced angiotensinogen and MCP-1 expression in a bidirectional manner in primary cultured mesangial cells. *Journal of the Renin-Angiotensin-Aldosterone System* **2020**, *21*(3), p.1470320320946527.

Perfetto, L.; Briganti, L.; Calderone, A.; Cerquone Perpetuini, A.; Iannuccelli, M.; Langone, F.; Licata, L.; Marinkovic, M.; Mattioni, A.; Pavlidou, T. and Peluso, D. SIGNOR: a database of causal relationships between biological entities. *Nucleic acids research* **2016**, *44(D1)*, D548-D554.

Ries, C.H.; Cannarile, M.A.; Hoves, S.; Benz, J.; Wartha, K.; Runza, V.; Rey-Giraud, F.; Pradel, L.P.; Feuerhake, F.; Klaman, I.; others. Targeting tumor-associated macrophages with anti-CSF-1R antibody reveals a strategy for cancer therapy. *Cancer cell* **2014**, *25*, p.846–859.

Satou, R., Miyata, K., Gonzalez-Villalobos, R.A., Ingelfinger, J.R., Navar, L.G. and Kobori, H. Interferon-γ biphasically regulates angiotensinogen expression via a JAK-STAT pathway and suppressor of cytokine signaling 1 (SOCS1) in renal proximal tubular cells. *The FASEB Journal* **2012**, *26*(5), p.1821-1830.

Schioppa, T.; Uranchimeg, B.; Saccani, A.; Biswas, S.K.; Doni, A.; Rapisarda, A.; Bernasconi, S.; Saccani, S.; Nebuloni, M.; Vago, L.; Mantovani, A. Regulation of the chemokine receptor CXCR4 by hypoxia. *The Journal of experimental medicine* **2003**, *198(9)*, p.1391-1402.

Solinas, G.; Germano, G.; Mantovani, A.; Allavena, P. Tumor-associated macrophages (TAM) as major players of the cancer-related inflammation. *Journal of leukocyte biology* **2009**, *86(5)*, p.1065-1073.

Solinas, G.; Schiarea, S.; Liguori, M.; Fabbri, M.; Pesce, S.; Zammataro, L.; Pasqualini, F.; Nebuloni, M.; Chiabrando, C.; Mantovani, A.; others. Tumor-conditioned macrophages secrete migration-stimulating factor: a new marker for M2-polarization, influencing tumor cell motility. *The Journal of Immunology* **2010**, *185*, p.642–652.

Szondy, Z. and Pallai, A. Transmembrane TNF-alpha reverse signaling leading to TGF-beta production is selectively activated by TNF targeting molecules: Therapeutic implications. *Pharmacological research* **2017**, *115*, p.124-132.

ten Hacken, E. and Burger, J.A. Microenvironment interactions and B-cell receptor signaling in Chronic Lymphocytic Leukemia: Implications for disease pathogenesis and treatment. *Biochimica et Biophysica Acta (BBA)-Molecular Cell Research* **2016**, *1863*(3), p.401-413.

Thompson, C.D., Matta, B. and Barnes, B.J. Therapeutic targeting of IRFs: pathway-dependence or structure-based?. *Frontiers in Immunology* **2018**, *9*, p.2622.

Van Dyken, S.J. and Locksley, R.M. Interleukin-4-and interleukin-13-mediated alternatively activated macrophages: roles in homeostasis and disease. *Annual review of immunology* **2013**, *31*, p.317-343.

Wu, W.K., Llewellyn, O.P., Bates, D.O., Nicholson, L.B. and Dick, A.D. IL-10 regulation of macrophage VEGF production is dependent on macrophage polarisation and hypoxia. *Immunobiology* **2010**, *215*(9-10), p.796-803.

Zhong, Z., Wen, Z. and Darnell, J.E. Stat3: a STAT family member activated by tyrosine phosphorylation in response to epidermal growth factor and interleukin-6. *Science* **1994**, *264*(5155), p.95-98.
